# Tip-Enhanced Raman Images of Realistic Systems through Ab Initio Modeling

**DOI:** 10.1021/acsnano.5c16052

**Published:** 2026-02-09

**Authors:** Krystof Brezina, Yair Litman, Mariana Rossi

**Affiliations:** † 375070 Max Planck Institute for the Structure and Dynamics of Matter, Luruper Chaussee 149, 22761 Hamburg, Germany; ‡ 28308 Max Planck Institute for Polymer Research, Ackermannweg 10, 55128 Mainz, Germany; § Yusuf Hamied Department of Chemistry, University of Cambridge, Lensfield Road, Cambridge CB2 1EW, U.K.

**Keywords:** tip-enhanced Raman spectroscopy, normal-mode imaging, surface systems, first-principles calculations, density-functional theory

## Abstract

Tip-enhanced Raman spectroscopy (TERS) is a powerful method for imaging vibrational motion and chemically characterizing surface-bound systems. Theoretical simulations of TERS images often consider systems in isolation, ignoring any substrate support, such as metallic surfaces. Here, we show that this omission leads to deviations from experimentally measured data, as demonstrated by simulations with a new finite-field periodic formulation of first-principles simulation of TERS spectra that can address extended systems. We show that TERS images of defective MoS_2_ monolayers calculated using cluster models are qualitatively different from those calculated when accounting for the periodicity of the substrate. For Mg­(II)-porphine on Ag(100), a system for which a direct experimental comparison is possible, these simulations prove to be crucial for explaining the spatial variation of TERS intensity patterns and allow us to uncover fundamental principles of TERS spectroscopy. We explain how and why surface interactions affect images of out-of-plane vibrational modes much more strongly than those of in-plane modes, thereby providing an important tool for the future interpretation of these images in more complex systems.

## Graphical abstract

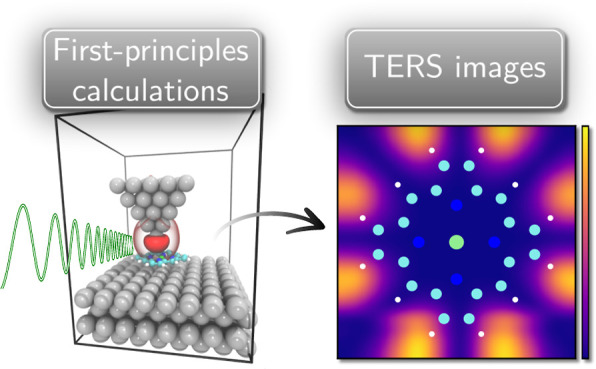

Raman spectroscopy is a well established tool to elucidate the atomic structure, the atomic composition and the vibrational motion of matter in the gas phase or the condensed phase. Obtaining sufficient intensity of the scattered signal relies on the existence of a large number of molecules or amount of the material.^1^ Moreover, due to the diffraction limit, it is impossible to obtain signals from spatially resolved regions in the subnanometer range with typical visible light wavelengths. These properties prevent the acquisition of signals coming from single-molecules, defects or impurities at surfaces.

Such drawbacks can be overcome with tip-enhanced Raman spectroscopy^2–4^ (TERS). This method borrows concepts from surface-enhanced Raman spectroscopy^5^ and relies primarily on the enhancement of the Raman signal by localized plasmonic resonances.^6^ Specifically in the case of TERS, these are created by design at the junction formed between the metal surface and an atomically sharp metallic tip by the action of an external radiation.^7^ This strong localization of the electromagnetic field at the nanoscale junction is the basis for the extreme spatial sensitivity of TERS that allows one to achieve subnanometer resolution and record Raman scattering from individual parts of molecules.^8–11^

TERS is a complementary technique to more established scanning probe methods such as scanning tunneling microscopy (STM), atomic force microscopy (AFM), and their various adaptations. By inheriting the chemical specificity of Raman spectroscopy, TERS enables the identification of the chemical structure of complex molecules. Over its two decades of existence, TERS has been employed to identify edge structures in 2D materials,^12^ measure interfacial strain,^13^ determine local temperatures,^14^ probe optical properties of localized defects,^15–17^ characterize self-assembled monolayers,^18,19^ sequence DNA,^20^ investigate surface-mediated and electrochemical reactions at the nano scale,^21–23^ investigate local phonons in twisted bilayer systems^24^ and, of course, for single-molecule imaging.^10,11^

In the case of molecular adsorbates, additional enhancement mechanisms arise beyond plasmonic effects. For instance, the chemical interaction of the molecule with the underlying surface can give rise to an additional enhancement known as chemical enhancement.^25–27^ The scattered signal therefore contains contributions from the supporting surface to varying degrees. On the one hand, these contributions can be exploited to study the specific properties of the interface and of surface–molecule interactions, but on the other hand, they can obfuscate certain molecular features.

Theoretical simulations of vibrational spectra represent an invaluable complement to their experimental measurement as a predictive and interpretative means.^28^ While for standard (i.e., far-field) Raman spectroscopy, the simulation approaches are well established,^29–32^ for TERS the situation becomes more complex owing to the presence of the highly nonhomogeneous plasmonic near field. Appropriate simulation approaches are a matter of active development.^10,11,25,33–36^ Existing methodologies have been generally successful in capturing the correct symmetries and shapes of the TERS images,^10,11,34^ however, an analysis of the literature shows that a method that obtains qualitative agreement with a broader range of experimental data is still lacking. For instance, Lee et al. in their pioneering TERS study of Co­(II)-tetraphenylporphyrin^11^ faithfully matched the experimental 1156 cm^–1^ asymmetric hydrogen bending mode image, but had qualitatively less success with the “Maltese-cross” mode image at 730 cm^–1^, both simulated with a methodology that considered the isolated molecule in the gas phase. In their discussion, the authors suggest the chemical interaction with the surface and the electronic screening as the primary culprits.

Motivated by these observations, we show in this paper that such shortcomings can be rectified by including the metal surface and the system’s periodicity from first principles. To this end, we develop a simulation methodology inspired by previous work by some of us,^37^ which can fully account for both atomistic description of the plasmonic near fields (see the overview in Figure 1) and, unlike the original method,^37^ extended metallic surfaces and periodic systems in general within first-principles density-functional theory (DFT) simulations. The technical details of the employed methodology can be found in Methods and in Sections S1–S4 of the Supporting Information. We employ this methodology to study TERS signals from defects and adsorbates as listed in Figure 1 and discussed in detail below.

**Figure 1:**
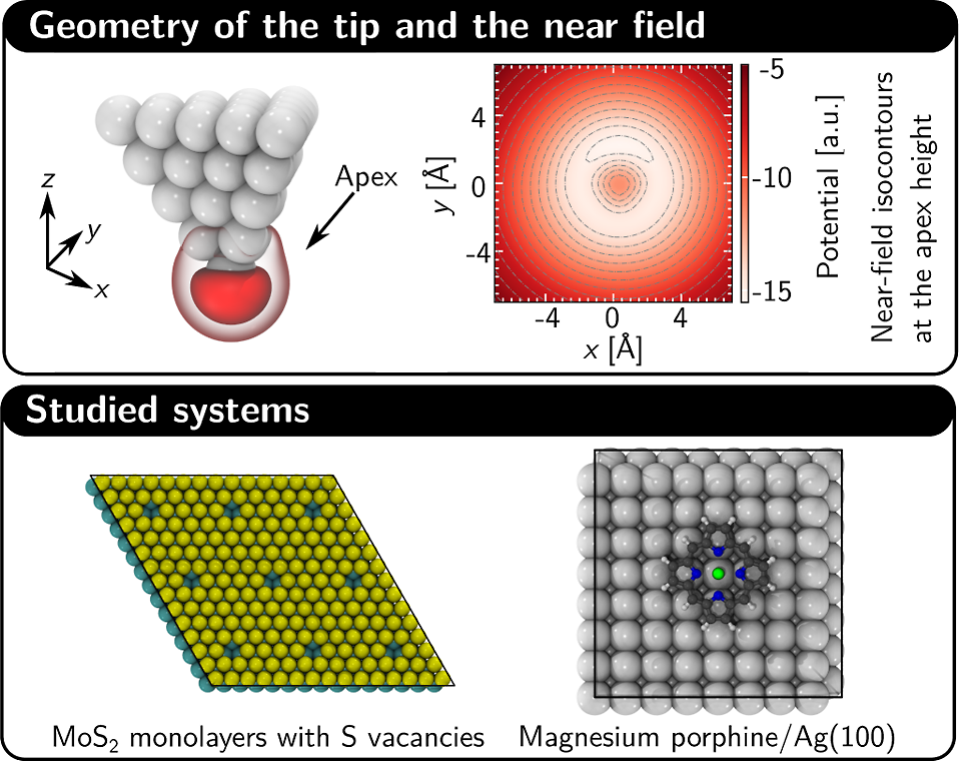
Top row (left to right): the geometry of the Ag tip including the spatial distribution of the atomistic near field (in red; darker shades correspond to a higher intensity contour) and a horizontal cut through the potential at the level of the apex that illustrates the deviation of the near field from central symmetry. The tip geometry and the near field potential are adopted from the “Tip A” geometry presented in refs 37, and 50 (see Methods and Section S11 of the Supporting Information for details). Bottom row (left to right): a bird's-eye view of the surface systems studied in this work including an MoS_2_ monolayer with 4% sulfur vacancy concentration and magnesium­(II) porphine (MgP)/Ag(100). All systems are treated under Born–von-Kármán boundary conditions and the boundary of the unit cell in each system is shown in black.

As we will show below, because the method we present here is able to address vastly more realistic systems while maintaining first-principles accuracy of the local-field distribution and of the TERS cross sections, we are able to study physical effects that change TERS image patterns and that had not been previously considered. These are effects related to larger spatial extent of vibrational modes, screening due to the metallic surfaces, and sensitivity to molecular adsorption height. The direct comparison to experimental data for MgP/Ag(100) provides a more stringent quality control of the underlying approximations in the theory, confirms the predictive capabilities of this method, and teaches us about the electronic effects that shape TERS images.

## Results

We benchmarked our methodology and implementation for the same system addressed in ref 37, namely the tetracyanoethylene (TCNE) molecule adsorbed on Ag(100). These benchmarks and simulations are shown in the Supporting Information, Sections S5 and S6 and provide three important conclusions. First, both methods yield identical TERS images for the TCNE molecule in isolation, showing consistency. Second, both methods show, again consistently, that adding some form of metallic substrate can profoundly impact the resulting TERS images. However, the third point is where they differ: the inclusion of the extended metallic substrate yields different TERS images compared to those obtained on a model cluster. Such isolation of the effect of the substrate’s periodicity shows the need for the current methodology in order to perform simulations comparable to experiments realized on extended systems.

As a first application of the method in this paper, we show the simulation of TERS images of defect-related vibrational modes in monolayer MoS_2_. In particular, we look for vibrational modes stemming from sulfur monovacancies, which represent the most abundant defect under usual experimental conditions.^38,39^ These calculations were previously attempted in ref 17 using a MoS_2_ flake instead of the periodic system. The results suggested a drop in TERS intensity around the vacancy site, but a clear interpretation was obfuscated by a large spatial variation of the TERS intensity across the flake due to the finite size and asymmetry of the system. We employ the proposed simulation approach for TERS imaging of the A_1_^′^ vibration in periodic monolayers, which produces a strong signal in TERS due to its out-of-plane character.

In Section S7 of the Supporting Information, we present the one-dimensional TERS and Raman spectra for the pristine and defective MoS_2_ monolayers across the frequency spectrum. We find that the pristine case is dominated by a signal at 397 cm^–1^ that can be unequivocally attributed to the A_1_^′^ out-of-plane vibration. The presence of defects leads to the appearance of a lower-intensity shoulder on the right side of the A_1_^′^ peak at 400 cm^–1^. This shoulder corresponds to defect-induced Raman-active vibrations, which have been observed both experimentally^15^ and computationally^40^ and form the so-called vibrational D band. In the following, we discuss the TERS images that correspond to the pristine and defective A_1_^′^ vibrations and the D band.

As shown in Section S8 of the Supporting Information, in the pristine monolayer, because of the perfectly concerted motion of the S atoms, the simulated image mirrors the geometry of this vibration and exhibits a very uniform intensity across the system. In fact, the minute intensity variations around the S atoms serve as a probe of the accuracy of our simulations, which we prove to be at least on the order of $10^{-4}\;e^{2}{Å}^{2}V^{-2}$ . This accuracy is only achievable with tight electronic-structure convergence criteria (see Section S2 for details).

The presence of defects lowers the symmetry of the system and effectively folds different phonon branches. As a consequence, multiple Γ-point modes appear within the smaller Brillouin zone in the wavenumber range corresponding to the pristine A_1_^′^ vibrational band. Here, we show in Figure 2 the TERS images of the modes that correspond to the highest Raman intensity in this wavenumber range (A_1_^′^, 396.3 cm^–1^, Figure 2A) and its right shoulder (D, 400.4 cm^–1^, Figure 2B), as calculated for a 5 × 5 unit cell (see Section S2 of the Supporting Information for details). This corresponds to a finite vacancy concentration of 4% with a defect-to-defect distance of 15.9 Å (cf. Figure 1, bottom left snapshot). Such defect concentrations are experimentally relevant and have been reported, for example, in large-scale TERS imaging of WS_2_ layers.^15^ As depicted in Figure 2, both vibrations exhibit a *C*_3_ symmetry axis centered at the vacancy and maintain the general out-of-plane character of the motion of the sulfur atoms.^40^ However, the motion becomes more complex as the vibrational direction of the S atoms is no longer collinear, and certain Mo atoms also partake in it when the vacancy is present. The corresponding TERS images feature a pattern that appears as a consequence of the defect. The A_1_^′^ mode displays a lower Raman intensity region centered at the defect site surrounded by a ring of higher intensity that clearly shows a *C*_3_ symmetry; similarly, the D band exhibits a trefoil-like symmetry centered at the vacancy. Obtaining such symmetry is only possible in a periodic system, or would necessitate flakes much larger than the characteristic length-scale of the spectral signal and that would not break the 3-fold symmetry of this system.

**Figure 2:**
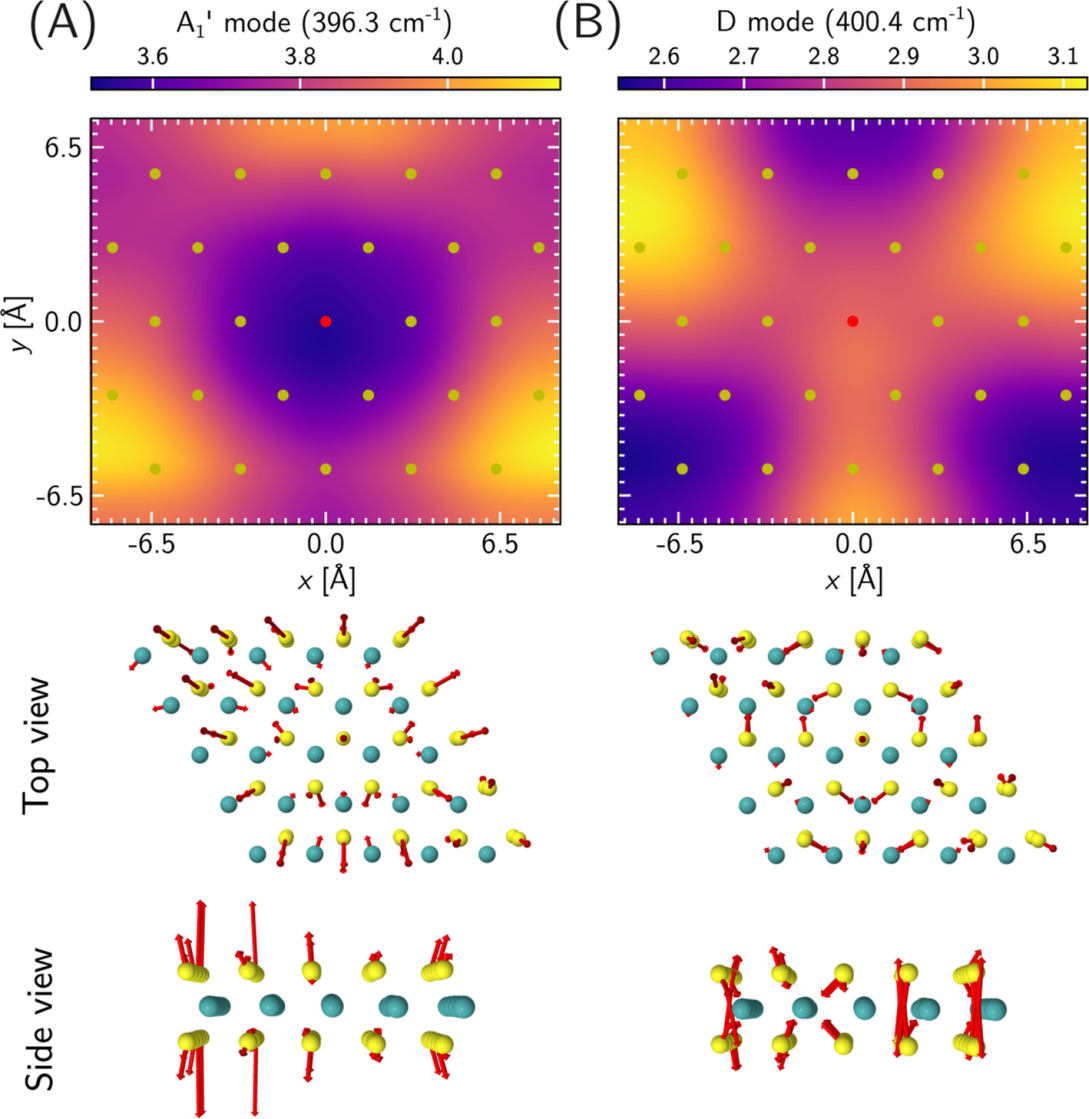
TERS imaging of the Raman-active defect-related equivalent of the A_1_^′^ (panel A) and D (panel B) vibrations in an MoS_2_ monolayer containing sulfur monovacancies. The top row shows the calculated TERS images. The positions of the top-layer sulfur atoms are given by the yellow circles and the location of the defect at (0, 0) Å is marked by the red dot. The TERS intensity is shown in the units of $10^{1}e^{2}{Å}^{2}V^{-2}$ . The snapshots show a top view and a side view of the vibrations on a 5 × 5 unit cell. In these snapshots, the sulfur atoms are shown in yellow, the molybdenum atoms in turquoise and the Cartesian atomic components of the normal mode vector as red arrows.

None of the defective modes shows a decay to the pristine A_1_^′^ symmetry away from the vacancy within the unit-cell size we considered. This suggests a long–range interaction between the defects at the nanometer scale and the emergence of collective vibrational states at finite defect concentrations that will render their spectroscopic fingerprint a complex function of the concentration and spatial distribution of the defects. While the experimental TERS imaging of vacancy defects in MoS_2_ at a subnanometer resolution remains a challenge,^16,41^ a simulation of an extended periodic system sets a reasonable starting point for a potential interpretation of such experiments.

We continue by presenting simulations of TERS images of the MgP/Ag(100) system, which uncovers principles underlying TERS image patterns. The symmetrical structure of this system, the well-defined adsorption geometry, the planar structure of MgP, and the availability of high-quality experimental data^10^ make this system particularly suitable for validating our approach and for elucidating the role of various electronic-structure changes on TERS images. Upon adsorption on the Ag(100) substrate, the MgP molecule only undergoes a negligible deviation from its gas-phase planar *D*_4h_ symmetry and adopts a position where the central Mg ion sits on top of an Ag atom in the surface layer of the metal slab. We inspect three representative normal modes across the frequency spectrum: a 193.4 cm^–1^ A_2u_ mode dominated by the out-of-plane vibration of the Mg atom (Figure 3, panel J), a 1359.9 cm^–1^ B_1g_ mode which captures the asymmetric breathing of the pyrrole rings (Figure 3, panel K) and, finally, a 3180.3 cm^–1^ A_2g_ asymmetric hydrogen stretching mode (Figure 3, panel L). All of these modes exhibit a rich spatial variation of the TERS signal as demonstrated by the experimental images in panels G–I of Figure 3, which were originally published by Zhang et al. in ref 10 (see Section S9 of the Supporting Information for additional details). Specifically, the A_2u_ mode shows the highest intensity above the central Mg atom with weak tails reaching toward the bridge CH groups; the B_1g_ mode has a distinct four-peak structure with maxima around the pairs of distal carbon atoms of the pyrrole rings; the A_2g_ mode shows an 8-peak pattern located on the pyrrole hydrogen atoms.

**Figure 3:**
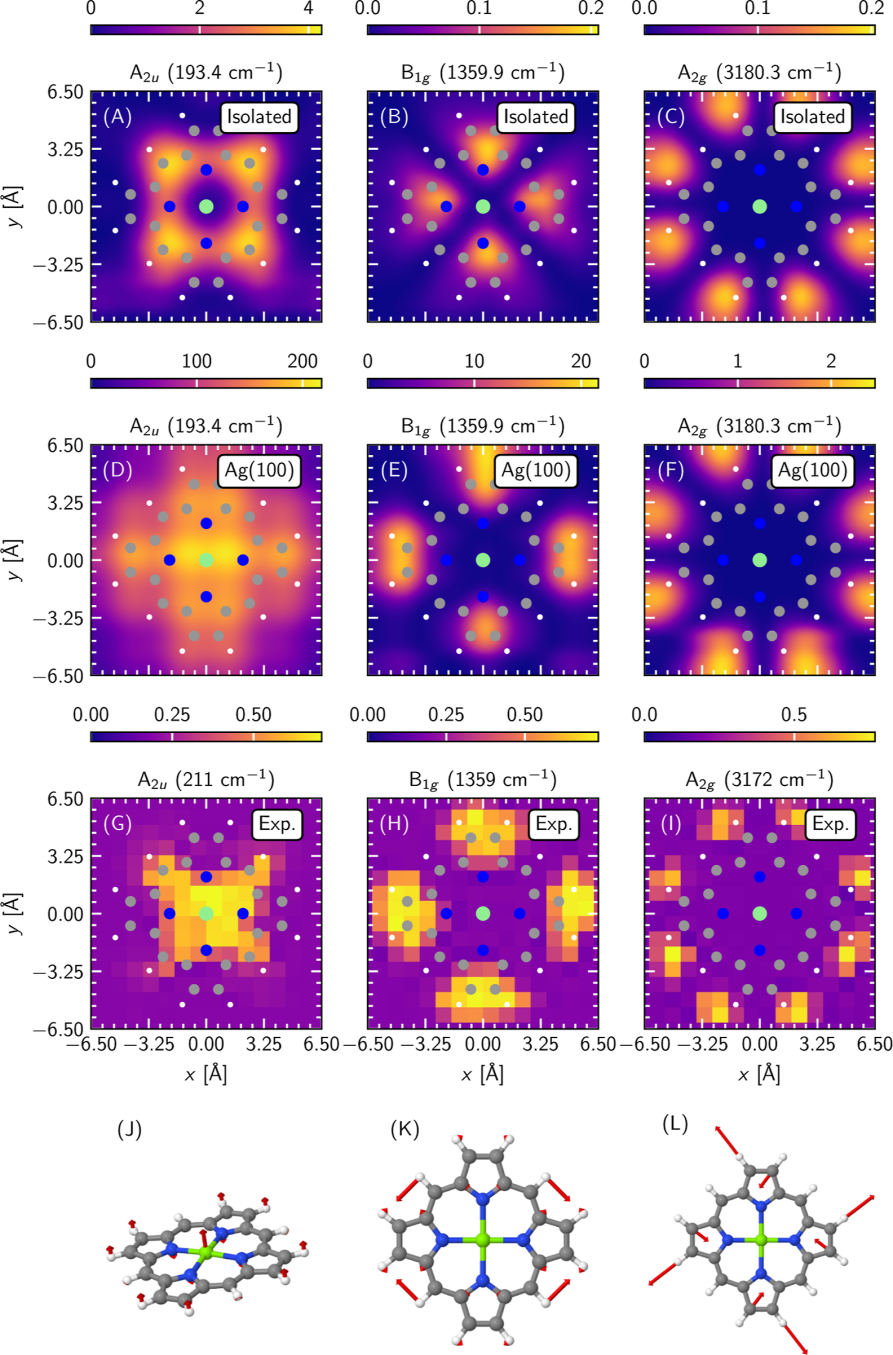
Simulation of TERS images of selected vibrational modes of MgP/Ag(100). Panels (A–C): Images obtained in the gas phase, however, using displacements calculated on the silver surface. Panels (D–F): Images obtained on the explicit silver surface. In all panels (A–F) the corresponding color bars show TERS intensities in the units of $10^{4}e^{2}{Å}^{2}V^{-2}$ , which is proportional to Raman intensity. Panels (G–I): Experimental TERS images of the studied modes, as reported in ref 10. The data was adapted from the original publication as detailed in Section S9 of the Supporting Information and the maxima of the intensity patterns were normalized to unity. Panels (J–L): Snapshots of the corresponding vibrational modes of MgP/Ag(100). The silver surface was removed for clarity and the red arrows show the atomic components of the Cartesian normal mode vectors. In all panels, the following color-coding of atomic species applies: Mg green, C gray, N blue, H white.

An important point to highlight before we discuss the TERS images in more detail is that all of the simulated images of molecules on surfaces (for example, see also the B_2_ mode of TCNE/Ag(100) in panel E of Figure S5) are affected to a varying degree by asymmetry. When there is no reason for such asymmetry to be present due to the geometry of the scattering subsystem, such as in the case of the systems presented in this paper, the only source of remaining asymmetry is the atomistic description of the tip potential. This potential is inherently asymmetric with respect to the symmetry of the molecular systems (both TCNE and MgP alike) since it bears an imprint of its original skewed trigonal-pyramidal silver cluster geometry^37^ (see the horizontal cut through the atomistic near field used in our calculations in Figure 1). In our calculations, the tip is positioned such that it breaks the symmetry of the *y* = 0 mirror plane; it is therefore fully consistent with the fact that all of our observed asymmetry manifests along the *y*-axis. We have confirmed this hypothesis explicitly by fitting a dipolar potential corresponding to the *z*-direction to the full tip potential. This fit has a *C*_*∞*_ axis and does not break any underlying molecular symmetries. As demonstrated in Section S10 of the Supporting Information, using this dipolar field to recreate the image of the B_1g_ MgP/Ag(100) mode creates a fully symmetric image. This finding opens an intriguing debate about how the tip geometry translates into the (a) symmetry of experimental images as the atomistic shape of the tip is typically not known. In turn, one could gain the ability to infer the shape of the tip by inspecting the shapes of the measured image after having first mapped out the relationship between the two computationally. We have additionally inspected and discussed other tip shape effects on the MgP B_1g_ TERS image in Section S11 of the Supporting Information.

In order to understand the origin of the TERS patterns of MgP/Ag(100), we first calculate the TERS images of the MgP molecule in the gas phase and show the obtained results in panels (A–C) of Figure 3. It is clear that in certain cases, the gas-phase approximation can give rise to images that are very close to the experiment, such as the A_2g_ mode displayed in panel C. However, this is not true in general. The 4-peak image of the B_1g_ mode (panel B) is described correctly only from a qualitative perspective but the peak positions are incorrect: the gas-phase image shows peak maxima around the pyrrole nitrogen atoms, whereas the experiment has peaks located around the distal pyrrole carbon atoms. The gas-phase simulation of the A_2u_ mode (panel A) is incorrect even from a qualitative viewpoint, as the image shows a pronounced minimum at the location of the central Mg atom where the experiment shows the highest intensity. Once again, we thus find that the gas-phase approximation is inaccurate as a means of comparison to surface-bound experimental data and shows alterations that depend on the nature of the specific vibrational mode in question and cannot be known a priori.

Now we turn our attention to the images simulated with the inclusion of an atomistic Ag(100) periodic surface as shown in panels D–F of Figure 3. These results show a significantly improved agreement with the experiment in all studied modes. The A_2g_ mode (panel F), which was already well described in the gas phase, retains this quality on the surface. The peaks of the B_1g_ mode image (panel E) are now positioned consistently with the experimental data. Finally and perhaps most interestingly, the surface simulation (panel D) reproduces the region of high intensity in the middle of the A_2u_ mode image observed experimentally (panel G), in contrast to the gas-phase simulation (panel A). Therefore, it allows us to link this intensity with the surface–molecule interaction and illustrates the active involvement of the metal substrate in the shaping of TERS images. At the same time that this simulation captures the buildup of TERS intensity above the Mg atom, it also fails to reproduce the tails of the experimental peaks extending toward the bridge CH groups. These two observations deserve further discussion.

We start by explaining why the shape of the image in Figure 3 panel D does not match more faithfully the shape of the experimental image reported in panel G. We find that the shape of the resulting image for this mode depends on the equilibrium surface–molecule separation distance. Computationally, this distance depends strongly on the choice of the DFT functional and, in particular, of the employed dispersion correction as we show in Section S12 of the Supporting Information for a systematic set of commonly used DFT functionals. Indeed, we find that screened many-body van-der-Waals corrections^42^ (MBD-NL) increase the molecule–surface distance by 0.21 Å, in comparison to the pairwise Tkatchenko–Scheffler (TS) dispersion^43^ used in most calculations in this paper. The puckering of the molecule remains virtually unchanged. Indeed, we explored different functionals and van der Waals correction combinations (see Figure S11 in Section S12 of the Supporting Information), concluding that the functionals regarded as most accurate yield a flat optimized structure and farther adsorption distances.

Taking this into consideration, we have recalculated the A_2u_ TERS image with the PBE/MBD-NL optimized geometry (Figure 4A). We find that this image is in better agreement with the experimental shape, demonstrating that the binding distance is a relevant criterion that depends on the DFT choice and that can change the TERS image of this mode. For this system and experimental setup, effects that are not included in our simulations, such as vibrational anharmonicity, charge transfer between the tip and the molecule, and phenomena beyond the ground-state DFT description are expected to be of minor or negligible relevance. This observation suggests that TERS images of specific modes could be indirectly used to determine surface–molecule distances of single molecules, which is often hard to measure in experiment.

**Figure 4:**
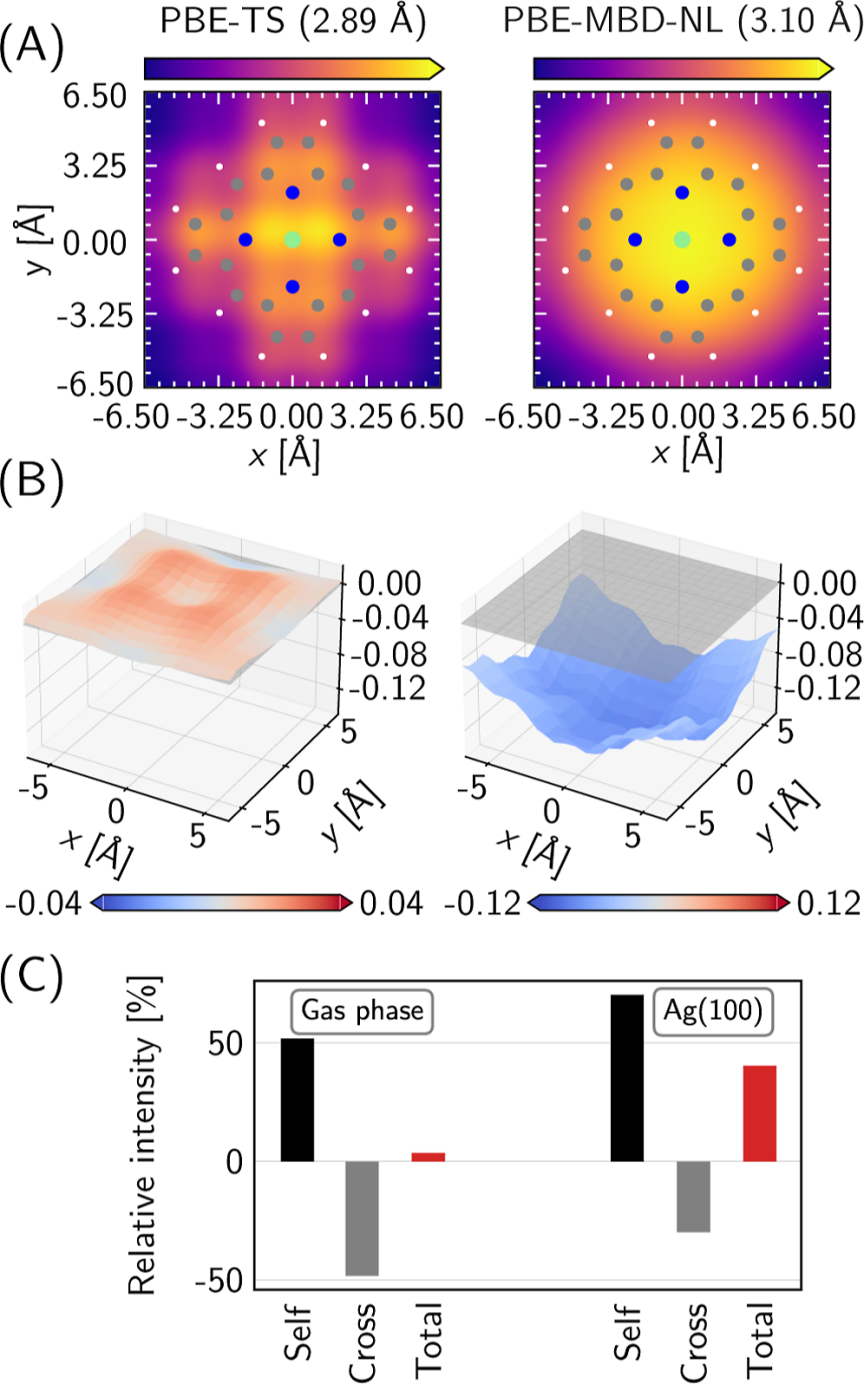
Further analysis of the shape and intensity patterns in the A_2u_ mode of MgP/Ag(100). Panel (A): The left plot shows the original simulated PBE/TS result from Figure 3D; the right plot shows the image of the same mode calculated on a geometry optimized at the PBE/MBD-NL. Both panels show the employed level of theory and the equilibrium surface–molecule distance in above the color bars. The direction of increase of the TERS intensity (*I*_*zz*_) is shown by the oriented color bar. Panel (B): The TERS amplitude *A*_*zz*_ calculated in the gas phase (left) and on the surface (right) shown as a 3D surface and its value is given in each plot by the color bars in the units of $eÅV^{-1}$ . The gray plane marks zero magnitude. The employed color map ranges from negative (darker blue) to positive (darker red) with the point of zero value in white. Panel (C): The decomposition of the TERS intensity into self-and cross terms (see eq 3) shows the near complete cancellation of the terms in the gas phase and incomplete cancellation on the surface.

We then continue with an analysis of how the surface acts to dramatically alter the TERS image of some modes (such as the A_2u_ mode of MgP) and to leave others practically unaltered. As explained in the Methods section, under suitable approximations the TERS intensity can be calculated as

1 $$I_{zz}\left(\omega_{k},R_{tip}\right)\propto {\left[\frac{\partial \alpha_{zz}^{local}\left(R_{tip}\right)}{\partial Q_{k}}\right]}^{2}\equiv A_{zz}^{2}\left(\omega_{k},R_{tip}\right)$$

where α_*zz*_^local^(**R**_tip_) is the *zz*-component of the tip-position **R**_tip_ dependent local polarizability and *Q*_k_ is the normal coordinate of a vibration with characteristic frequency ω_k_. To gain further insight, we inspect the quantity *A*_*zz*_, which describes how the polarizability changes with the vibration and carries a sign and, therefore, can be called the TERS amplitude. This quantity is shown for the gas-phase and the surface-bound MgP A_2u_ vibration in Figure 4B. The gas-phase amplitude is almost exclusively positive and features the central minimum at the Mg atom surrounded by the four peaks over the bridge C atoms. In the surface-bound case, the general shape of the amplitude in the vicinity of the molecule remains the same: the Mg atom features a minimum and the bridge C atoms feature four regions of higher intensity. However, the amplitude is negative, which leads to the emergence of the central maximum in the corresponding TERS image.

The variations of the amplitude around the molecule are a nontrivial result of the specific interaction of the vibrating molecule with the plasmonic near field and the underlying metal surface. The fundamental cause of the relative sign change in *A*_*zz*_, however, does not depend on the details of the near-field. We find that the primary source of this effect is the screening of the induced dipole by the Ag(100) substrate as the molecular vibration takes place. We explicitly demonstrate this by tracking the changes in electron density response as a function of vibrational displacement and provide additional discussion in Section S13 of the Supporting Information.

Predicting the quantitative scale of the change in *A*_*zz*_ requires first-principles modeling as it depends on the specific chemistry of the system. However, we can infer from the presented *A*_*zz*_ plots in Figure 4B that the sign change is consistent throughout the whole lateral extent of the image, and thus points to an effect that is independent of the distribution of the near field. As such, it would also be noticeable in standard Raman intensity, and this could be used as a useful qualitative marker to predict the presence of such screening effects. This effect can be understood as follows: In the gas-phase, the molecular distortion along this normal mode leads to an increase in the corresponding polarizability tensor component, meaning that response of the electronic density is enhanced when the molecule is distorted. When the molecule is adsorbed, instead, the electrons of the surface act to screen this effect and lead to a decrease in the electronic density response when the molecule is displaced along the same normal mode. The near field only enhances this effect.

In essence, only modes that have a nonzero Raman intensity along the scattering direction can exhibit this sign-change effect. This is the case for the A_2u_ (out-of-plane) mode in MgP/Ag(100). The remaining two in-plane modes shown in Figure 3 have vanishing Raman intensities along *z* owing to symmetry and, therefore, these modes can only exhibit changes in their intensity enhancement patterns that do not change the symmetry nodal planes. We note that this reasoning also explains why the TERS pattern in the B_1_ mode of TCNE (Figure S5A vs S5D) on the Ag(100) surface is less altered than what was observed on an Ag(100) cluster where the symmetry is broken. Finally, we note in passing that our calculations work with tip models of limited size, which translates into an arbitrary scaling of the near-field potential^36^ (see the Methods section). We find that such scaling, within the limits of linear polarization, maps into a scaling of the overall TERS intensity, but does not affect the relative sign change in *A*_*zz*_ between the gas-phase and the surface systems.

Finally, we attempt to gain a deeper understanding of local and nonlocal contributions to TERS intensity enhancement patterns. The intensity patterns are often qualitatively described as having a large magnitude where the atomic motion within a normal mode is large.^44^ We check this assumption by performing a decomposition of the TERS intensity into atomic terms. We can use a chain rule to write

2 $$\begin{array}{ll} A_{zz}\left(\omega_{k},R_{tip}\right) & =\frac{\partial \alpha_{zz}^{local}\left(R_{tip}\right)}{\partial Q_{k}}\\ & =\sum_{i}\frac{\partial \alpha_{zz}^{local}\left(R_{tip}\right)}{\partial q_{i}}a_{k,i}\\ & =\sum_{i}A_{ik}\left(R_{tip}\right), \end{array}$$

where **a**_*k*_ is the Cartesian *k*th normal mode vector and *q*_*i*_ are Cartesian and $A_{ik}$ was defined on the last line. Therefore, when calculating the TERS intensity according to eq 1, there will be terms that involve only displacements on the same atom and cross terms that multiply displacements on different atoms

3 $$I_{zz}\left(\omega_{k},R_{tip}\right)=I_{zz}^{self}\left(\omega_{k},R_{tip}\right)+I_{zz}^{cross}\left(\omega_{k},R_{tip}\right)$$

where

4 $$I_{zz}^{self}\left(\omega_{k},R_{tip}\right)=\sum_{i}{\left[A_{ik}\left(R_{tip}\right)\right]}^{2}$$

and

5 $$I_{zz}^{cross}\left(\omega_{k},R_{tip}\right)=\sum_{i}\;\sum_{j\neq i}A_{ik}\left(R_{tip}\right)A_{jk}\left(R_{tip}\right).$$

While the self-terms are strictly non-negative, the cross terms can assume negative values as they are products of terms that do not necessarily have the same sign.

Such a decomposition can provide an atomistic perspective on the emergence of the observed sign change of *A*_*zz*_ (eq 2) as discussed in detail in Section S14 of the Supporting Information. Both *I*_*zz*_^self^ and *I*_*zz*_^cross^ are shown for the tip positioned right above the Mg atom for the A_2u_ MgP mode in Figure 4C. In the gas phase, the *I*_*zz*_^cross^ almost perfectly cancels the *I*_*zz*_^self^, leading to the very small intensity observed at the origin despite the fact that the Mg atom has by far the largest amplitude of motion in this mode. At variance, *I*_*zz*_^cross^ on the surface has a noticeably smaller magnitude, which results in an incomplete cancellation of the two and, consequently, the observed nonzero intensity peak at the origin. This perspective shines a new light on the interpretation of TERS images: the presence of cross terms connecting different atoms can lead to the emergence of nontrivial patterns beyond those reflecting the normal-mode geometry. Further discussion and interpretation of this spectral decomposition is provided in Section S15 of the Supporting Information.

## Conclusions

In conclusion, the results we have presented led to a much deeper understanding regarding the interpretation of TERS images from experiments and simulations. The initial benchmarks (Section S6 of the Supporting Information) and simulations we performed on defective MoS_2_ monolayers proved that approximating the surface as a cluster (or omitting it altogether in the case of molecules in a so-called gas-phase calculation) is inaccurate, leading to images that contain artifacts. A proper handling of periodicity is crucial for an accurate modeling. The simulations and direct comparison to experiment of the MgP/Ag(100) system led to new insights into the origins of the shape of TERS images, including its dependence on the surface–molecule distance and, importantly, how it is impacted by the screening effects of the surface. We find that such screening plays a fundamental role in the overall amplitude of the vibrational normal modes featuring out-of-plane changes of polarization (such as the A_2u_ mode in MgP). In addition, we provided a quantitative estimate of the nonlocal contributions to intensity patterns of TERS images, thus showing that the common assumption that TERS images have larger magnitudes where the atomic motion is larger is not always valid.

While in this work we consider only molecules on metallic substrates or free-standing monolayers, we remark that the presented method is equally suitable for other systems where the interaction with the substrate is strong, regardless of the electronic properties of the substrate. In particular, MoS_2_ monolayers are normally supported by some material, which, depending on its character, can change the electronic structure and vibrational properties of MoS_2_.^17,45–47^ In fact, because negatively charged vacancies (e.g., on Au support^17,48^ ) undergo a Jahn–Teller distortion, we expect TERS to be exquisitely sensitive to such symmetry breaking. The method we developed is already able to simulate TERS spectra of such systems, and a deeper study of the impact of the supporting substrate on TERS fingerprints of transition-metal dichalcogenide monolayers will be of great value to the community.

We identify the main approximations of the current methodology as the following: (1) limitation to relatively small tip models for the plasmonic near-field distribution; (2) the use of static perturbations that preclude the treatment of resonant TERS signals; (3) the assumption of nonoverlapping electronic densities between tip and molecule or substrate, that precludes the treatment of the contact regime; (4) lack of reradiation enhancement mechanisms;^49^ (5) limitation to the surface-normal component of the polarizability tensor. Augmenting the method along any of these directions will increase its realm of application, including areas showing interesting new spectroscopic behavior.^50,51^ While some of these extensions require new physics to be incorporated in the model, others, such as larger tip sizes and resonant responses, would more directly benefit from modern machine-learning models capable of dealing with such complex electronic responses. It is, nevertheless, very refreshing to see that the method presented in this work is very well suited to model many current TERS experimental setups and unravel new understanding of these measurements for systems of essentially arbitrary complexity.

The understanding achieved in this work was only possible due to the methodology we developed. We presented a computationally efficient, first-principles framework for calculating TERS spectra and images on periodic substrates, using finite-field perturbations. The method is implemented in the FHI-aims electronic structure software^52^ and thus widely available to the community, together with tutorial repositories and automating workflows (see Methods). Even though we have presented TERS intensity patterns obtained within a harmonic approximation for nonresonant Raman scattering, the methodology is very flexible and it is readily possible to couple it with dynamical methods that allow the study of anharmonic vibrational motion, of reactive events, and the inclusion of nuclear quantum effects.

## Methods

We briefly outline the main underlying principles of the employed methodology in the following paragraphs. A fully first-principles solution of TERS would call for a real-time propagation of the quantum system that includes both the scattering subsystem and the tip under the influence of the oscillating far-field perturbation. While this is technically possible, the high computational cost of explicitly propagating the electronic degrees of freedom limits applicability to the smallest scattering subsystems.^53–55^ Therefore, most commonly used approaches rely on a combination of a phenomenological localization of the near field and either a polarizable force-field description of the surface,^33,34^ or omit the surface altogether and simulate the molecules as isolated entities.^10,11^

With the aim of accounting for the substrate in efficient, first-principles TERS simulations, some of us^37^ have formulated the following approximate theory. The time-dependent problem of the whole interacting system including the surface, the molecule (or any spatially localized chemical environment) and the tip under the influence of an external periodic electromagnetic radiation is transformed into a static problem with the perturbed Hamiltonian

6 $$\begin{array}{ll} Ĥ & ={Ĥ}_{0}^{sc}+{\mathrm{\Phi ̂}}_{0}\left(R_{tip}\right)\\ & +E_{z}\left\{-{\mathrm{\mu ̂}}_{z}^{sc}+{\left[\frac{\partial \tilde{\hat{\Phi }}\left(\omega_{p};R_{tip}\right)}{\partial E_{z}}\right]}_{E_{z}=0}\right\}. \end{array}$$

In this expression, ${Ĥ}_{0}^{\left(sc\right)}$ represents the Hamiltonian terms pertaining to the scattering subsystem (e.g., molecule and its supporting surface or defect center in a material), *E*_*z*_ is the intensity of the component perpendicular to the surface plane of the incoming far field, ${\mathrm{\mu ̂}}_{z}^{sc}$ is the *z*-component of the dipole operator of the scattering subsystem, **R**_tip_ is the position of the tip and ${\mathrm{\Phi ̂}}_{0}$ is the unperturbed electrostatic scalar field of the tip. The key quantity $\tilde{\hat{\Phi }}\left(\omega_{p}\right)$ represents the Fourier component at the plasmon excitation frequency ω_p_ of the oscillating electrostatic potential Φ̂(t) of the isolated tip under the influence of the incoming radiation. The quantity ${\mathrm{\Phi ̂}}_{0}$ can be neglected at a price of introducing a reasonably small error (as shown in Section S3 of the Supporting Information), leading to a significant computational speed up.

Importantly, the quantity Φ̂(t) is calculated for an isolated tip using real-time time-dependent (TD) DFT^56^ and the derivative with respect to *E*_*z*_ is obtained numerically by performing simulations at several field strengths. This must only be done once for a given tip geometry^37^ and the spatial distribution of the derivative of the resulting Fourier-transformed quantity $\tilde{\hat{\Phi }}\left(\omega_{p}\right)$ is stored and used off the shelf for arbitrary scattering subsystems. In this spirit, we rely on the existing near field distributions presented in ref 37 and available in the repository of this project.^57^ We refer the reader to ref 37 for details of the TDDFT simulations performed using the Octopus software.^58^

Equation 6 is valid under three reasonable assumptions: (a) a tip-molecule distance large enough that there is no electronic density overlap, charge transfer, or current between the tip and the molecule, meaning that the interaction is dominated by electrostatics. (b) *E*_*z*_ weak enough that the induced tip polarization varies linearly with its intensity, (c) nonresonant Raman scattering so that the contributions from electron dynamics can be disregarded and only the static problem is relevant. These approximations render our method not directly applicable to the STM-TERS regime of measurement, which relies on a current flowing between the tip and the sample simultaneously at the time of the Raman measurement. Note that we limit ourselves to the *z*-components of all vector quantities: these are experimentally relevant as most measurements are realized with the near field oriented approximately parallel to the surface normal direction (chosen to be *z*), with detection in the backscattering regime.^7^

Using Ĥ in eq 6, density-functional perturbation theory^32,59^ (DFPT) can be applied in order to calculate the relevant *zz*-component of the spatially dependent polarizabilty tensor α_*zz*_(**R**_tip_). Under the harmonic approximation, the corresponding Raman intensity is given by

7 $$I_{zz}\left(\omega_{k},R_{tip}\right)\propto {\left[\frac{\partial \alpha_{zz}^{local}\left(R_{tip}\right)}{\partial Q_{k}}\right]}^{2}$$

Here, *Q*_*k*_ is the normal coordinate of the *k*th vibrational mode with eigenfrequency ω_*k*_.

A drawback of this methodology is that the real-space DFPT formulation is not easily applicable to periodic systems under nonhomogeneous electric perturbations, in particular if the surface is metallic.^32^ The present work reformulates this methodology for use in periodic systems, which is achieved by a replacement of the DFPT-based step with a finite-field calculation. Specifically, through a self-consistent solution of Ĥ in eq 6 under Born–von-Kármán boundary conditions, one obtains an electron density ρ­(**r**; **R**_tip_) and, subsequently a *z*-component of a dipole moment via a real-space integration

8 $$\mu_{z}^{sc}\left(R_{tip}\right)=\int_{unitcell}drz\rho \left(r;R_{tip}\right)$$

This dipole component is always physically meaningful as the *z*-direction is (effectively) aperiodic in the slab geometry. A dipole-correction is always applied. The next key step relies on the application of a finite homogeneous electric field of magnitude Δ*E*_*z*_ (which turns on the whole perturbation term including the near-field coupling in eq 6) to calculate the *zz*-component of the polarizability tensor through a finite difference as

9 $$\alpha_{zz}^{local}\left(R_{tip}\right)=\frac{\partial \mu_{z}^{sc}\left(R_{tip}\right)}{\partial E_{z}}=\frac{\Delta \mu_{z}^{sc}\left(R_{tip}\right)}{\Delta E_{z}}$$

since we are ensuring being in the regime of linear polarization, the finite difference is exact. In the expression above, Δ*μ*_*z*_^sc^ represents the induced dipole moment

10 $$\begin{array}{ll} \Delta \mu_{z}^{sc}\left(R_{tip}\right) & =\mu_{z}^{sc}\left(R_{tip},E_{z}=\Delta E_{z}\right)\\ & -\mu_{z}^{sc}\left(R_{tip},E_{z}=0\right) \end{array}$$

Once the polarizabilities are known, one can proceed to the calculation of TERS images under the harmonic approximation using eq 7. Note that under open boundary conditions, the two formulations are identical as we demonstrate in Section S5 of the Supporting Information. We have implemented this new approach in FHI-aims and provide a thorough description of the specific computational details in Section S2 of the Supporting Information. The implementation is available free of charge for academic purposes in https://fhi-aims.org and is documented in the current FHI-aims manual. A Python-based infrastructure allowing for, among other functionalities and examples, an automated generation of TERS scanning grids for FHI-aims near-field embedding calculations is publicly available in our GitHub repository.^57^ For completeness, we briefly mention the essential technical parameters of the performed TERS simulations (see Section S2 for more details). We described the electronic structure PBE^60^ density functional equipped with the Tkatchenko–Scheffler dispersion correction.^43^ All Hessian calculations were performed using a two-point finite difference with a Cartesian displacement of atomic positions of 5 × 10^–3^ Å, while relying on the frozen-surface approximation. The gas-phase calculations of TCNE and MgP were accomplished with the surface-adsorbed geometry of each molecule and the corresponding normal modes. For the TERS calculations, we employed a tip height of 4 Å and a 20 × 20 pixels lateral scan grid for the surface–molecule systems and a 12 × 12 grid for the MoS_2_ systems. The total scanned areas were 1.0, 1.69, and 2.65 nm^2^ for TCNE, MgP and MoS_2_, respectively.

## Supplementary Material



## Data Availability

The data supporting the findings of this study are available in Zenodo at 10.5281/zenodo.18457490.

## Notes

Theoretical derivation of the principal equations; Computational details; Numerical tests and validations; TCNE/Ag(100) benchmark; 1D Raman and TERS spectra of all systems; pristine MoS_2_ monolayer TERS imaging; Reconstruction of the experimental TERS images; Additional discussion of tip geometry, symmetry and placement; Discussion of the effect of surface–molecule binding distance on TERS; Additional insights into the origin of the shapes of TERS images (PDF) (PDF)

## References

[1] Craig, D. P. ; Thirunamachandran, T. Molecular Quantum Electrodynamics: An Introduction to Radiation Molecule Interactions; Dover Publications, Inc., 1984.

[2] Pozzi E. A., Goubert G., Chiang N., Jiang N., Chapman C. T., McAnally M. O., Henry A. I., Seideman T., Schatz G. C., Hersam M. C., Duyne R. P. V. (2017). Ultrahigh-Vacuum
Tip-Enhanced Raman
Spectroscopy. Chem. Rev..

[3] Shao F., Zenobi R. (2019). Tip-Enhanced Raman Spectroscopy:
Principles, Practice,
and Applications to Nanospectroscopic Imaging of 2D Materials. Anal. Bioanal. Chem..

[4] Cai Z. F., Kumar N., Zenobi R. (2023). Probing on-Surface
Chemistry at the
Nanoscale Using Tip-Enhanced Raman Spectroscopy. CCS Chem..

[5] Han X. X., Rodriguez R. S., Haynes C. L., Ozaki Y., Zhao B. (2022). Surface-Enhanced
Raman Spectroscopy. Nat. Rev. Methods Primers.

[6] Kneipp K., Kneipp H., Itzkan I., Dasari R. R., Feld M. S. (1999). Ultrasensitive
Chemical Analysis by Raman Spectroscopy. Chem.
Rev..

[7] Zhang Z., Sheng S., Wang R., Sun M. (2016). Tip-Enhanced Raman
Spectroscopy. Anal. Chem..

[8] Zrimsek A. B., Chiang N., Mattei M., Zaleski S., McAnally M. O., Chapman C. T., Henry A. I., Schatz G. C., Van Duyne R. P. (2017). Single-Molecule
Chemistry with Surface- and Tip-Enhanced Raman Spectroscopy. Chem. Rev..

[9] Steidtner J., Pettinger B. (2008). Tip-Enhanced
Raman Spectroscopy and Microscopy on Single
Dye Molecules with 15 Nm Resolution. Phys. Rev.
Lett..

[10] Zhang Y., Yang B., Ghafoor A., Zhang Y., Zhang Y. F., Wang R. P., Yang J. L., Luo Y., Dong Z. C., Hou J. G. (2019). Visually Constructing the Chemical
Structure of a Single
Molecule by Scanning Raman Picoscopy. Natl.
Sci. Rev..

[11] Lee J., Crampton K. T., Tallarida N., Apkarian V. A. (2019). Visualizing Vibrational
Normal Modes of a Single Molecule with Atomically Confined Light. Nature.

[12] Huang T.-X., Cong X., Wu S.-S., Lin K.-Q., Yao X., He Y.-H., Wu J.-B., Bao Y.-F., Huang S.-C., Wang X., Tan P.-H., Ren B. (2019). Probing the Edge-Related
Properties of Atomically Thin MoS_2_ at Nanoscale. Nat. Commun..

[13] Rahaman M., Rodriguez R. D., Plechinger G., Moras S., Schüller C., Korn T., Zahn D. R. T. (2017). Highly Localized Strain in a MoS_2_/Au Heterostructure Revealed by Tip-Enhanced Raman Spectroscopy. Nano Lett..

[14] Cirera B., Wolf M., Kumagai T. (2022). Joule Heating
in Single-Molecule
Point Contacts Studied by Tip-Enhanced Raman Spectroscopy. ACS Nano.

[15] Kato R., Moriyama T., Umakoshi T., Yano T., Verma P. (2022). Ultrastable
Tip-Enhanced Hyperspectral Optical Nanoimaging for Defect Analysis
of Large-Sized WS_2_ Layers. Sci. Adv..

[16] Jorio A., Nadas R., Pereira A. G., Rabelo C., Gadelha A. C., Vasconcelos T. L., Zhang W., Miyata Y., Saito R., Costa M. D. D., Cançado L. G. (2024). Nano-Raman
Spectroscopy of 2D Materials. 2D Mater..

[17] Akkoush A., Litman Y., Rossi M. (2024). A Hybrid-Density Functional
Theory
Study of Intrinsic Point Defects in MX_2_ (M = Mo, W; X =
S, Se) Monolayers. Phys. Status Solidi A.

[18] Toccafondi C., Picardi G., Ossikovski R. (2016). Molecular Bending at the Nanoscale
Evidenced by Tip-Enhanced Raman Spectroscopy in Tunneling Mode on
Thiol Self-Assembled Monolayers. J. Phys. Chem.
C.

[19] Zhang P., Chen L., Sheng S., Hu W., Liu H., Ma C., Liu Z., Feng B., Cheng P., Zhang Y., Chen L., Zhao J., Wu K. (2022). Melamine Self-Assembly
and Dehydrogenation on Ag(111) Studied by Tip-Enhanced Raman Spectroscopy. J. Chem. Phys..

[20] Han Y., Dong L., Zhu L. Y., Hu C. R., Li H., Zhang Y., Zhang C., Zhang Y., Dong Z. C. (2024). Real-Space
Spectral Determination of Short Single-Stranded DNA Sequence Structures. J. Am. Chem. Soc..

[21] Fiocco A., Pavlic A. A., Kanoufi F., Maisonhaute E., Noël J.-M., Lucas I. T. (2024). Electrochemical Tip-Enhanced Raman
Spectroscopy for the Elucidation of Complex Electrochemical Reactions. Anal. Chem..

[22] Sabanés N. M., Ohto T., Andrienko D., Nagata Y., Domke K. F. (2017). Electrochemical
TERS Elucidates Potential-Induced Molecular Reorientation of Adenine/Au(111). Angew. Chem., Int. Ed..

[23] Bhattarai A., El-Khoury P. Z. (2019). Nanoscale Chemical Reaction Imaging at the Solid-Liquid
Interface via TERS. J. Phys. Chem. Lett..

[24] Gadelha A. C., Ohlberg D. A. A., Rabelo C., Neto E. G. S., Vasconcelos T. L., Campos J. L., Lemos J. S., Ornelas V., Miranda D., Nadas R., Santana F. C., Watanabe K., Taniguchi T., van Troeye B., Lamparski M., Meunier V., Nguyen V.-H., Paszko D., Charlier J.-C., Campos L. C. (2021). Localization
of Lattice Dynamics in Low-Angle Twisted Bilayer Graphene. Nature.

[25] Jensen L., Aikens C. M., Schatz G. C. (2008). Electronic
Structure Methods for
Studying Surface-Enhanced Raman Scattering. Chem. Soc. Rev..

[26] Gieseking R. L., Ratner M. A., Schatz G. C. (2017). Theoretical
Modeling of Voltage Effects
and the Chemical Mechanism in Surface-Enhanced Raman Scattering. Faraday Discuss..

[27] Itoh T., Procházka M., Dong Z.-C., Ji W., Yamamoto Y. S., Zhang Y., Ozaki Y. (2023). Toward a New Era of SERS and TERS
at the Nanometer Scale: from Fundamentals to Innovative Applications. Chem. Rev..

[28] Tuckerman, M. E. Statistical Mechanics: Theory and Molecular Simulation; Oxford University Press Inc., 2010.

[29] Marx, D. ; Hutter, J. Ab Initio Molecular Dynamics: Basic Theory and Advanced Methods; Cambridge University Press, 2009.

[30] Wan Q., Spanu L., Galli G. A., Gygi F. (2013). Raman Spectra of Liquid
Water from Ab Initio Molecular Dynamics: Vibrational Signatures of
Charge Fluctuations in the Hydrogen Bond Network. J. Chem. Theory Comput..

[31] Marsalek O., Markland T. E. (2017). Quantum Dynamics
and Spectroscopy of Ab Initio Liquid
Water: the Interplay of Nuclear and Electronic Quantum Effects. J. Phys. Chem. Lett..

[32] Shang H., Raimbault N., Rinke P., Scheffler M., Rossi M., Carbogno C. (2018). All-Electron, Real-Space Perturbation
Theory for Homogeneous Electric Fields: Theory, Implementation, and
Application within DFT. New J. Phys..

[33] Payton J. L., Morton S. M., Moore J. E., Jensen L. (2014). A Hybrid Atomistic
Electrodynamics-Quantum Mechanical Approach for Simulating Surface-Enhanced
Raman Scattering. Acc. Chem. Res..

[34] Liu P., Chulhai D. V., Jensen L. (2017). Single-Molecule Imaging Using Atomistic
Near-Field Tip-Enhanced Raman Spectroscopy. ACS Nano.

[35] Cançado L. G., Beams R., Jorio A., Novotny L. (2014). Theory of Spatial Coherence
in Near-Field Raman Scattering. Phys. Rev. X.

[36] Duan S., Tian G., Luo Y. (2024). Theoretical and Computational
Methods
for Tip- and Surface-Enhanced Raman Scattering. Chem. Soc. Rev..

[37] Litman Y., Bonafé F. P., Akkoush A., Appel H., Rossi M. (2023). First-Principles
Simulations of Tip Enhanced Raman Scattering Reveal Active Role of
Substrate on High-Resolution Images. J. Phys.
Chem. Lett..

[38] Li S., Nishimura T., Maruyama M., Okada S., Nagashio K. (2023). Experimental
Verification of SO_2_ and S Desorption Contributing to Defect
Formation in MoS_2_ by Thermal Desorption Spectroscopy. Nanoscale Adv..

[39] Ascrizzi E., Nalesso M., Marana N. L., Milotti G., Granozzi G., Agnoli S., Ferrari A. M. (2025). Defect
Engineering in MoS_2_ Monolayers on Au(111): Insights from
Combined Experimental and Theoretical
Approaches. J. Phys. Chem. C.

[40] Zhao S., Lu M., Xue S., Yan L., Miao P., Hang Y., Wang X., Liu Z., Wang Y., Tao L., Sui Y., Wang Y. (2019). A Se Vacancy
Induced Localized Raman Mode in Two-Dimensional
MoSe_2_ Grown by CVD. arXiv.

[41] An H., Li J., Liu Y., Xu P., Han S., Liu Y., Chen S., Li S.-Y., Lin C., Pan A. (2024). Tip-Enhanced
Raman Spectroscopy of Monolayer MoS_2_ on Au(111). J. Phys. Chem. C.

[42] Hermann J., Tkatchenko A. (2020). Density Functional Model for Van Der Waals Interactions:
Unifying Many-Body Atomic Approaches with Nonlocal Functionals. Phys. Rev. Lett..

[43] Tkatchenko A., Scheffler M. (2009). Accurate Molecular
Van Der Waals Interactions from
Ground-State Electron Density and Free-Atom Reference Data. Phys. Rev. Lett..

[44] Duan S., Tian G., Luo Y. (2016). Visualization
of Vibrational Modes
in Real Space by Tip-Enhanced Non-Resonant Raman Spectroscopy. Angew. Chem., Int. Ed..

[45] Klein J., Kerelsky A., Lorke M., Florian M., Sigger F., Kiemle J., Reuter M. C., Taniguchi T., Watanabe K., Finley J. J., Pasupathy A. N., Holleitner A. W., Ross F. M., Wurstbauer U. (2019). Impact of
Substrate Induced Band Tail States on the Electronic and Optical Properties
of MoS_2_. Appl. Phys. Lett..

[46] Chen W., Santos E. J. G., Zhu W., Kaxiras E., Zhang Z. (2013). Tuning the
Electronic and Chemical Properties of Monolayer MoS_2_ Adsorbed
on Transition Metal Substrates. Nano Lett..

[47] Simon J. R., Maksimov D., Lotze C., Wiechers P., Felipe J. P. G., Kobin B., Schwarz J., Hecht S., Franke K. J., Rossi M. (2024). Atomic-Scale Perspective on Individual Thiol-Terminated Molecules
Anchored to Single S Vacancies in MoS_2_. Phys. Rev. B.

[48] Tan A. M. Z., Freysoldt C., Hennig R. G. (2020). Stability of Charged Sulfur Vacancies
in 2D and Bulk MoS_2_ from Plane-Wave Density Functional
Theory with Electrostatic Corrections. Phys.
Rev. Mater..

[49] Ausman L. K., Schatz G. C. (2009). On the Importance
of Incorporating Dipole Reradiation
in the Modeling of Surface Enhanced Raman Scattering from Spheres. J. Chem. Phys..

[50] Cirera B., Litman Y., Lin C., Akkoush A., Hammud A., Wolf M., Rossi M., Kumagai T. (2022). Charge Transfer-Mediated
Dramatic Enhancement of Raman Scattering upon Molecular Point Contact
Formation. Nano Lett..

[51] Kumagai T., Miwa K., Cirera B. (2025). Point-Contact Tip-Enhanced
Raman
Spectroscopy: Picoscale Light–Matter Interactions within Plasmonic
Cavities. Nano Lett..

[52] Abbott J. W., Acosta C. M., Akkoush A., Ambrosetti A., Atalla V., Bagrets A., Behler J., Berger D., Bieniek B., Björk J., Blum V., Bohloul S., Box C. L., Boyer N., Brambila D. S., Bramley G. A., Bryenton K. R., Camarasa-Gómez M., Carbogno C., Caruso F. (2025). Roadmap on Advancements of the FHI-Aims Software Package. arXiv.

[53] Liu S., Bonafe F. P., Appel H., Rubio A., Wolf M., Kumagai T. (2023). Inelastic Light Scattering
in the Vicinity of a Single-Atom
Quantum Point Contact in a Plasmonic Picocavity. ACS Nano.

[54] Zhao L. L., Jensen L., Schatz G. C. (2006). Surface-Enhanced
Raman Scattering
of Pyrazine at the Junction between Two Ag_20_ Nanoclusters. Nano Lett..

[55] Jensen L., Zhao L. L., Schatz G. C. (2007). Size-Dependence
of the Enhanced Raman
Scattering of Pyridine Adsorbed on Ag_n_ (*n* = 2–8, 20) Clusters. J. Phys. Chem.
C.

[56] Yabana K., Bertsch G. F. (1996). Time-Dependent Local-Density
Approximation in Real
Time. Phys. Rev. B.

[57] Brezina, K. ; Rossi, M. Periodic TERS GitHub Repository of the SAbIA Group. https://github.com/sabia-group/periodic-ters (accessed Feb 2, 2026).

[58] Tancogne-Dejean N., Oliveira M. J., Andrade X., Appel H., Borca C. H., Le Breton G., Buchholz F., Castro A., Corni S., Correa A. A., De Giovannini U., Delgado A., Eich F. G., Flick J., Gil G., Gomez A., Helbig N., Hübener H., Jestädt R., Jornet-Somoza J. (2020). Octopus, a Computational
Framework for Exploring Light-Driven Phenomena
and Quantum Dynamics in Extended and Finite Systems. J. Chem. Phys..

[59] Baroni S., de Gironcoli S., Dal Corso A., Giannozzi P. (2001). Phonons and
Related Crystal Properties from Density-Functional Perturbation Theory. Rev. Mod. Phys..

[60] Perdew J. P., Burke K., Ernzerhof M. (1996). Generalized
Gradient Approximation
Made Simple. Phys. Rev. Lett..

[61] Brezina K., Litman Y., Rossi M. (2025). Explaining
Principles of Tip-Enhanced
Raman Images with Ab Initio Modeling. arXiv.

