# Supplementary material for: Tip-Enhanced Raman Images of Realistic Systems through Ab Initio Modeling
Source: ACS Nano. 2026 Feb 9;20(7):5550–60. doi: 10.1021/acsnano.5c16052 (PMC12947729; doi:10.1021/acsnano.5c16052)
Supplement: Supplementary file 1 [file nn5c16052_si_001.pdf]

Supporting Information for: Tip-Enhanced Raman Images of Realistic Systems Through Ab Initio Modeling

Krystof Brezina,[1] Yair Litman,[2,3] and Mariana Rossi[1,3]
[1)] *Max Planck Institute for the Structure and Dynamics of Matter, Luruper Chaussee 149, 22761 Hamburg, Germany*
[2)] *Max Planck Institute for Polymer Research, Ackermannweg 10, 55128 Mainz, Germany*
[3)] *Yusuf Hamied Department of Chemistry, University of Cambridge, Lensfield Road, Cambridge, CB2 1EW, United Kingdom*

(Dated: 6 February 2026)

S1. THEORETICAL BACKGROUND

In the following paragraph, we rewrite the derivation of Equation 6 presented in our previous work,[S1] in order to achieve a self-contained explanation of this implementation. Our approach starts with the full, interacting and time-dependent problem described by the dipole-approximation Hamiltonian

$$\hat{H}(t) = \hat{H}_0^{\mathrm{sc}} + \hat{H}_0^{\mathrm{tip}} - E_z \cos(\omega_{\mathrm{p}} t)\left(\hat{\mu}_z^{\mathrm{sc}} + \hat{\mu}_z^{\mathrm{tip}}\right) + \hat{V}(t), \quad \text{(S1)}$$

where $\hat{H}_0$ are the Hamiltonians of the unperturbed tip and the scattering (sc) subsystem, E_z is the magnitude of the electric far field component of the incoming laser radiation oscillating at the plasmonic frequency ω_{p}, $\hat{\mu}_z$ are the dipole operators of the two subsystems and $\hat{V}(t)$ is the interaction between them. Note that we limit ourselves to the z-components of all vector quantities: these represent the direction of the surface normal and are experimentally most relevant ones as TERS measurements are normally realized in the vertical back-scattering regime. Next, we reduce the above expression to a much simpler static perturbation problem through several reasonable assumptions. First, a tip-molecule distance large enough that there is no electronic density overlap, charge transfer, or current between the tip and the molecule, meaning that the interaction is dominated by electrostatics. This allows us to express the interaction only as the electrostatic (*i.e.*, Hartree) potential of the tip under the influence of $E_z \cos(\omega_{\mathrm{p}} t)$ in the position representation as

$$V(\mathbf{r}, t; \mathbf{R}_{\mathrm{tip}}) \approx \Phi(\mathbf{r}, t; \mathbf{R}_{\mathrm{tip}}) \equiv \frac{1}{4\pi\varepsilon_0} \int \mathrm{d}\mathbf{r}' \, \frac{\rho(\mathbf{r}, t; \mathbf{R}_{\mathrm{tip}})}{|\mathbf{r} - \mathbf{r}'|}. \quad \text{(S2)}$$

This is possible, because the tip density and the scattering-subsystem density do not overlap at large separations. Next, we assume that the incoming radiation is weak enough as to ensure the linear polarization regime. With that, we can Taylor-expand the field dependence of Φ to the first order around zero as follows

$$\Phi(\mathbf{r}, t; \mathbf{R}_{\mathrm{tip}}) \approx \Phi_0(\mathbf{r}; \mathbf{R}_{\mathrm{tip}}) + E_z \left[\frac{\partial \Phi(\mathbf{r}, t; \mathbf{R}_{\mathrm{tip}})}{\partial E_z}\right]_{E_z=0}. \quad \text{(S3)}$$

In addition, we assume that the time evolution of Φ is dominated by a single frequency, *i.e.*, that its Fourier transform can be well approximated as a Dirac δ:

$$\begin{aligned} &\left[\frac{\partial \tilde{\Phi}(\mathbf{r}, \omega; \mathbf{R}_{\mathrm{tip}})}{\partial E_z}\right]_{E_z=0} \\ &= \int_{-\infty}^{+\infty} \mathrm{d}t \; e^{-\mathrm{i}\omega t} \left[\frac{\partial \Phi(\mathbf{r}, t; \mathbf{R}_{\mathrm{tip}})}{\partial E_z}\right]_{E_z=0} \\ &\approx \left[\frac{\partial \tilde{\Phi}(\mathbf{r}, \omega_{\mathrm{p}}; \mathbf{R}_{\mathrm{tip}})}{\partial E_z}\right]_{E_z=0} \delta(\omega - \omega_{\mathrm{p}}), \end{aligned} \quad \text{(S4)}$$

which is reasonable in materials with a strong and isolated plasmonic response such as Ag. In turn, this allows us to simplify the time-dependence in Equation S3 to

$$\begin{aligned} \Phi(\mathbf{r}, t; \mathbf{R}_{\mathrm{tip}}) &\approx \Phi_0(\mathbf{r}; \mathbf{R}_{\mathrm{tip}}) \\ &+ E_z \cos(\omega_{\mathrm{p}} t) \left[\frac{\partial \tilde{\Phi}(\mathbf{r}, \omega_{\mathrm{p}}; \mathbf{R}_{\mathrm{tip}})}{\partial E_z}\right]_{E_z=0}. \end{aligned} \quad \text{(S5)}$$

These transformations allow us to split Equation S1 into separate expressions for the isolated tip and the scattering subsystem under the influence of an external potential. The time-dependent tip problem is solved using real-time TDDFT on the side and the key quantity $\tilde{\Phi}$ is numerically stored: the data format of our choice is a Gaussian Cube file. The Equation for the scattering subsystem then reads

$$\begin{aligned} H^{(\mathrm{sc})}(t) &= H_0^{(\mathrm{sc})} + \Phi_0(\mathbf{r}; \mathbf{R}_{\mathrm{tip}}) \\ &+ E_z \cos(\omega_{\mathrm{p}} t) \left\{ -\mu_z^{(\mathrm{sc})} + \left[\frac{\partial \tilde{\Phi}(\mathbf{r}, \omega_{\mathrm{p}}; \mathbf{R}_{\mathrm{tip}})}{\partial E_z}\right]_{E_z=0} \right\}. \end{aligned} \quad \text{(S6)}$$

The lack of hats over the operators in this expression implies assuming the position representation for all terms. Finally, we restrict ourselves to non-resonant Raman scattering which allows us to formulate the corresponding time-independent problem

$$\begin{aligned} H^{(\mathrm{sc})} &= H_0^{(\mathrm{sc})} + \Phi_0(\mathbf{r}; \mathbf{R}_{\mathrm{tip}}) \\ &+ E_z \left\{ -\mu_z^{(\mathrm{sc})} + \left[\frac{\partial \tilde{\Phi}(\mathbf{r}, \omega_{\mathrm{p}}; \mathbf{R}_{\mathrm{tip}})}{\partial E_z}\right]_{E_z=0} \right\} \end{aligned} \quad \text{(S7)}$$

which is directly addressable by static DFT and was implemented in FHI-aims[S2] with a Gaussian cube file input for the spatially varying components of the external tip potential for the work in Ref. S1. That implementation only worked for open boundary conditions. In this contribution, we have instead derived and implemented a finite-field version for the calculation of the perturbation term and added it to the infrastructure in FHI-aims. The calculation of the corresponding relevant polarizability components and the subsequent TERS intensity is described in detail in the main text. We finalize by noting that while this approach qualitatively captures the so-called antenna effects, it does not account for light–matter hybridization between the tip and substrate, nor does it include reradiation effects, which may become important under certain conditions.

S2. COMPUTATIONAL WORKFLOW

In this section, we provide a comprehensive overview of the computational details, including geometry relaxation, vibrational analysis and the TERS imaging simulations.

Geometry preparation and Hessian calculation

All geometries were combined using tools provided by the ASE Python package[S3] and optimized within the FHI-aims software.[S4] In order to correctly apply the Born–von-Kármán periodic boundary conditions, the surface systems need to be large enough to accommodate the numerically tabulated tip potential within a single unit cell. For the Ag(100) slab used in the simulations of TCNE and MgP, this means a size of 4 vertically organized layers of 8×8 atoms, leading to the total of 256 Ag atoms inside a periodic cell with a horizontal side length of 23.51 Å. We have tested the Ag slab thickness convergence in terms of vibrational modes and frequencies against a corresponding 6-layer slab to find a maximum deviation in vibrational wavenumber between the 4- and 6-layer systems of $\sim$20 cm^{-1}. For the MoS_2 systems, we used 15×15 unit cells with a total of 675 atoms, a lateral side length of 47.7 Å and the angle between the lateral unit cell vectors of 120°. A 50 Å-thick vacuum layer was added to each side of all slabs.

Regarding the electronic structure, we employed the *"light"* settings and the PBE generalized-gradient-approximation density functional[S5] augmented by the Tkatchenko–Scheffler (TS) dispersion correction[S6] for the TCNE and MgP systems. This setup has been used extensively to study molecular adsorption at surfaces, yielding reasonable adsorption geometries and vibrational frequencies.[S7,S8] Moreover, it allows us for a direct comparison with our previous work where it was also employed.[S1] In addition, we found that for the MoS_2 monolayers, especially those including vacancies, a much more converged electronic structure set up was needed to obtain well-behaved vibrational properties. For that reason, we used the *"tight"* basis set, increased the radial multiplier keyword[S4] to the value of 4 and the wave threshold keyword to 10^{-9} au. To ensure tight convergence of the electronic density, we employed a self-consistent cycle density convergence parameter of 10^{-7} ea_0^{-3} in all systems. The gas-phase systems were treated under open boundary conditions. The slabs were treated with a full 3D periodicity and a dipole correction[S9] was employed across the vacuum-containing direction (*i.e.*, z) to prevent spurious interactions between neighboring replicas. We stress that the dipole correction is necessary to yield the correct response behavior of the electronic structure of the systems under small applied electric fields. For the required slab sizes, we found it was sufficient to consider only Γ-point Kohn–Sham states.[S4,S10]

We found that converging the symmetries of the TERS images, especially the ones with low intensities, requires a very tight geometry optimization. For this reason, we employed a strict convergence criterion of 10^{-4} eVÅ^{-1} and relied on the Broyden–Fletcher–Goldfarb–Shanno (BFGS) minimizer[S11–S14] to relax our structures. The lengths of the lattice vectors of the Ag(100) slabs were first optimized without the TCNE and MgP adsorbates while keeping the angles fixed. Then, we deployed the molecules on the Ag surfaces and continued the optimization with a fixed unit-cell size to fully optimize the adsorbed configurations. During both optimization steps, we have kept the bottom two layers of the Ag slab constrained in their bulk geometry. For the MoS_2 slabs, we first optimized smaller slabs and later generalized to the aforementioned bigger systems for the TERS calculations. For the pristine monolayer, we optimized a 5×5 surface including the lattice vectors with fixed angles using a 3×3×1 Γ-centered k-point grid. For the defective system carrying a sulfur monovacancy, we continued the optimization in a fixed unit cell after removing a single sulfur atom from the optimized pristine monolayer.

Once we had access to relaxed geometries, we proceeded to the calculation of the Hessian matrices of the studied systems. To achieve this, we relied on the `Vibrations` module of ASE to generate Cartesian displacements and compute their energies and forces using an FHI-aims client with settings consistent with the ones described above. In all Hessian calculations, we employed Cartesian displacements of $5{\cdot}10^{-3}$ Å for each degree of freedom. To save computational time, we restricted the Hessian calculations in the case of the TCNE and MgP adsorbates on Ag(100) to the subspace of the configurational space that belong to the molecule. In those cases, the Hessian is well approximated by a block-diagonal structure with minimal vibrational couplings between the surface and the molecule. Therefore, the explicit surface must not necessarily *move* in the Hessian calculation, but it must still be *present* as it strongly affects the electronic structure and the geometry of the molecule. We have tested the validity of this approximation explicitly for MgP/Ag(100) by comparing the vibrational modes

and frequencies to a Hessian obtained with the first layer of the slab moving. We found that the maximum deviation in molecular vibrational wavenumber due to the frozen surface was ~20 cm^{-1}. No appreciable changes in vibrational motion were detected. For TCNE/Ag(100), this approximation was already employed and validated in Ref. S1. For the pristine and defective MoS_2 slabs, we calculated the Γ-point modes of a smaller 5×5 unit cell and extended the obtained A_1' mode (or the corresponding defective mode) to the full 15×15 system needed for the TERS calculation. In the case of the defective monolayer, this corresponds to a vacancy concentration of 4%.

Calculation of local polarizabilities

To accomplish the calculation of the TERS images presented in the main text, we rely exclusively on our FHI-aims implementation of the finite-field version of the electronic calculation in the presence of plasmonic near fields as described in Section S1. For practical reasons, the Φ_0 can be neglected as we show in Section S3. Our implementation expects the volumetric tip local field tabulated in a Gaussian cube format and our calculations rely on the exact same tip potential as used in our previous work,[S1] which was calculated using the Octopus software.[S15] No new real-time time-dependent DFT calculations were performed in this work.

We have implemented an FHI-aims workflow for the reading, interpolation and relative positioning of these cube files with respect to the scattering subsystem. Essentially, this means that a single FHI-aims control file is able to set up a full single-point electronic structure calculation including the tip local potential that yields a value of $\mu_z(\mathbf{R}_{\text{tip}})$ for a given tip position $\mathbf{R}_{\text{tip}}$ (the tip position is usually defined by the tip apex). Then, scanning over a grid of values of $\mathbf{R}_{\text{tip}}$ at two different values of E_z allows to use the finite-field formulation of local polarizabilities (Equation 9 of the main text) and Raman intensities (Equation 7 of the main text) thus yielding all information to construct a TERS image.

For this purpose, we employed for all systems a Cartesian displacement of the structures along normal mode vectors of length of $5 \cdot 10^{-3}$ Å and calculated the dipole moment z-components at field values of 0 and 10^{-1} V $Å^{-1}$ for the finite difference evaluation. For the TCNE in the gas phase and on Ag(100), we used a 20×20 pixel grid spanning from −5.0 to 5.0 Å in both lateral directions, for the MgP we used the same number of pixels, but spanning a larger area of −6.5 to 6.5 Å and for the MoS_2 slabs, we used 12×12 grid spanning −8.75 to 8.75 Å in the x-direction and −7.57 to 7.57 Å in the y-direction (*cf.* Fig. 2 for the corresponding monolayer orientation). For all TERS calculations, we set the distance between the tip and the scattering subsystem to 4 Å. For TCNE and MgP, this distance was defined between the tip apex and the molecular center of mass; for MoS_2 between the tip apex and the top S plane. For the gas-phase calculations of TCNE and MgP, we always keep the molecule in the surface-adsorbed geometry to meaningfully quantify the effect of the surface.

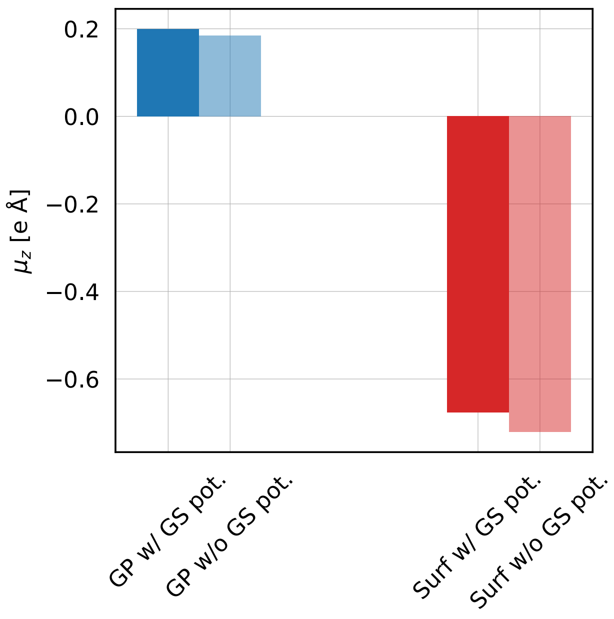

FIG. S1. The dipole moment of the TCNE molecule in the gas phase (GP) and on the Ag(100) surface calculated with or without the ground state tip potential (*cf.* second term of the right-hand side of Equation 6 of the main text). In all cases the magnitude of the applied homogeneous far field was set to 10^{-3} V $Å^{-1}$ and the derivative of the tip potential with respect to the far field was used as usual.

S3. NEGLECT OF GROUND-STATE HARTREE POTENTIAL TERM

For practical reasons, the TERS images in the main text were calculated while neglecting the $\Phi_0(\mathbf{r};\mathbf{R}_{\text{tip}})$ term in Equation 6 of the main text. The justification is that at tip–molecule distances relevant to our calculations, the ground state potential only has a minor overlap with the electron density of the scattering subsystem. On the other hand, the derivative of the potential with respect to the electric field extends spatially far below the tip apex and overlaps strongly with the electron density. In Fig. S1, we show the dipole moment z-component values calculated for the TCNE molecule in the gas phase and on Ag(100) with the tip located 4 Å above the center of the central C–C bond, where the tip is at its closest. The calculated value of the dipole moment only changes by 5–7% after including the ground state term. This implies that its contribution is more than an order of magnitude smaller that that of the potential derivative and neglecting it only introduces a minor effect, if any, on the resulting shape of the TERS images.

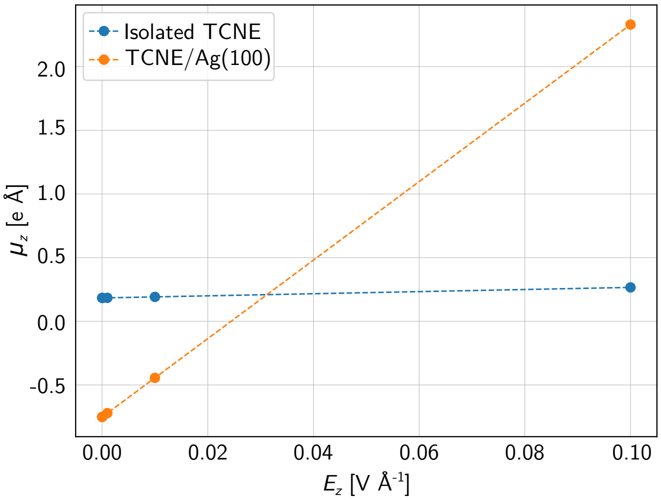

FIG. S2. The dipole moment of the isolated TCNE molecule (blue) and Ag(100)-bound TCNE (orange) in the presence of the tip near field at applied homogeneous electric fields ranging from 0 to 0.1 V Å^{-1}.

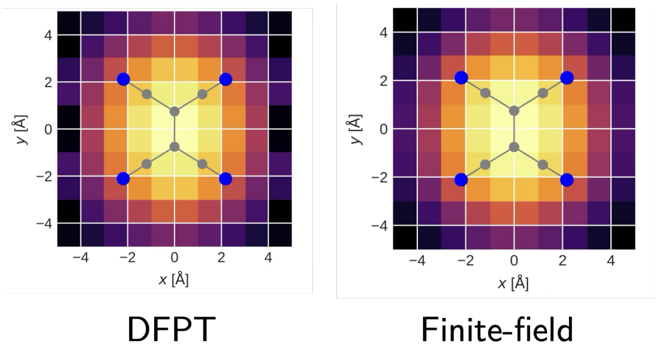

FIG. S3. The A_1 mode TERS image of gas-phase TCNE calculated using DFPT (left) and the FF-based approach (right). The latter is equivalent to Fig. S5C. The intensities are encoded through the color map, increase towards lighter colors and have the same range.

Note that the neglect of the ground state potential has a significant positive impact on the computational demands of our calculations. Once neglected, the $E_z = 0$ part of the calculation loses all its dependence on $\mathbf{R}_{\text{tip}}$. In turn, this means that the zero-field part of the calculation is essentially only two single-point dipole evaluations at the two displaced geometries. Therefore, for a TERS image with a grid of N^2 pixels, while the full calculation requires the total of $4N^2$ single-point calculations (N^2 for two displacements and two field strengths), the calculation neglecting the ground state only requires $2N^2 + 2$ single-points. Given the required size of the surfaces needed for these calculations, this can represent a significant speed up.

S4. VALIDITY OF THE LINEAR POLARIZATION REGIME

Another approximation behind Equation 6 of the main text is that the applied fields only polarize the system linearly. This allows us to use the first-order Taylor expansion in Equation S3. In Fig. S2, we show the dependence of μ_z on the magnitude E_z, for the TCNE molecule both in the gas phase and on Ag(100). Once again, the tip local field (only represented by the potential derivative with respect to E_z) is located 4 Å above the central C–C bond. For both systems, we are safely in the linear polarization regime up $E_z = 10^{-1}$ V Å^{-1}.

S5. BENCHMARK OF DFPT AND FINITE-FIELD CALCULATIONS FOR GAS-PHASE TCNE

We present a comparison of the present implementation using finite fields (FF) and the previous one of Ref. S1 using DFPT for a selected TERS image in Fig. S3. Both methods aim to calculate the zz-component of the polarizability tensor and are, therefore, equivalent. We show results for the A_1 mode of gas-phase TCNE, where we use the exactly same geometry and find that, within numerical accuracy given by the different nature of the two calculations, the obtained polarizabilities (and, consequently, TERS images) are indeed identical. However, we note again that only the FF-TERS implementation can be employed to periodic systems.

S6. THE IMPACT OF PERIODICITY ON TERS IMAGES OF TCNE ON AG(100)

We benchmark our methodology by revisiting tetracyanoethylene (TCNE) on Ag(100), a system that shows substrate effects on TERS images.[S1] The simulations presented in Ref. S1 were obtained by approximating the Ag(100) surface by an Ag cluster. The images produced for Ref. S1 are shown for comparison below, in Fig. S4, for completeness.

The TCNE molecule is fully planar in the gas phase, but bends its nitrogen atoms towards the surface upon adsorption, leading to a C_{2v} symmetric structure that is not disrupted by the surface when the molecule is at its optimal top adsorption site. Relying on the current simulation methodology described in Section S2 above, we simulated TERS images for the same modes as in the previous work, but considering the periodic system with the Ag(100) surface. Because we benchmarked our method as shown in Section S5, we are certain that any differences arising between these new simulations and the old ones are only due to the inclusion of periodicity. We

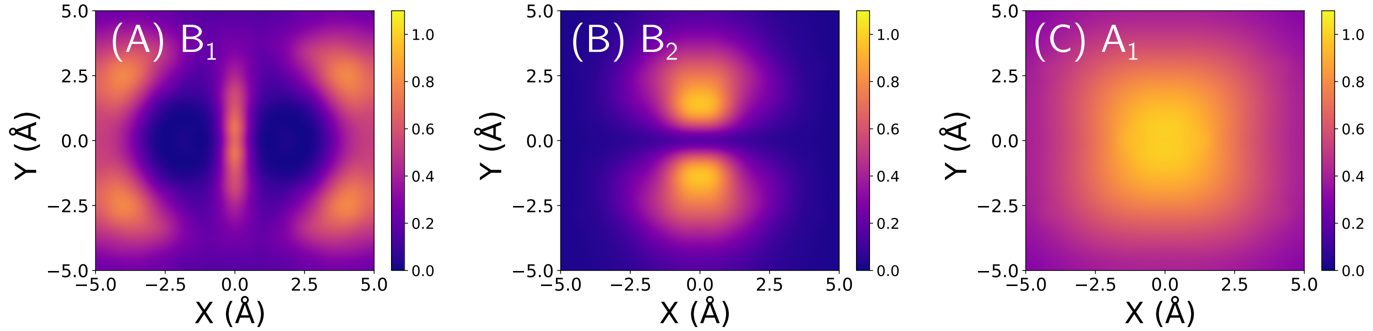

FIG. S4. TERS images of TCNE on a 4×4×3 Ag(100) cluster corresponding to the periodic Ag(100) surface images shown in Fig. S5A–C in Section S6. The intensities in this plot are normalized to the maximum intensity in each image. Adapted from Ref. S1.

considered a B_1 mode at 453.4 cm^{-1} (Fig. S5, panels A, E and I), a B_2 mode at 497.1 cm^{-1} (Fig. S5, panels B, F and J), an A_1 mode at 1246.0 cm^{-1} (Fig. S5, panels C, G and K) and an A_2 mode at 1293.0 cm^{-1} (Fig. S5, panels D, H and L). In order to allow a quantitative estimate of the effect of the surface, we used the vibrational modes calculated for the surface-bound system to create atomic displacements in the gas phase.

Both the A_1 and B_2 mode images are affected by the Ag(100) surface. The gas-phase TERS image of the A_1 mode is characterized by a single, broad peak positioned at the central C–C bond and spanning the whole TCNE molecule. Upon adsorption, the shape of the image is preserved, but the surface causes a significant enhancement of the Raman intensity by a factor of $\sim 10^3$. Interestingly, chemical enhancements are typically on the order of 10–100.[S16] We believe that the much larger enhancement observed here is related to the fact that TCNE is a strong electron acceptor that undergoes substantial charge transfer.[S1,S17]

In the case of the B_2 mode, the Raman intensity remains similar upon adsorption to Ag(100), but it is the shape of the image that changes noticeably. Specifically in the gas phase, the image exhibits a four-lobe pattern with peaks around the N atoms, whereas on the Ag(100) surface, the pattern is distinctly two-lobe with peaks more closely localized to the region of the C atoms that mainly partake in the vibration. Both findings are consistent with the previous results based on Ag clusters shown in Fig. S4.

However, the alteration of the TERS image of the B_1 mode between the gas phase and the Ag(100) cluster discussed in Ref. S1 is not reproduced on the periodic Ag(100) surface: compare Fig. S4A and Fig. S5E. Note that the only difference between these two calculations is the inclusion of periodicity. Hence, we attribute the previously observed effect in the B_1 mode to artifacts due to the finite size of the substrate and the asymmetry of the cluster. Finally, the A_2 mode retains its four-lobe character and only becomes ever-so-slightly more extended on the surface.

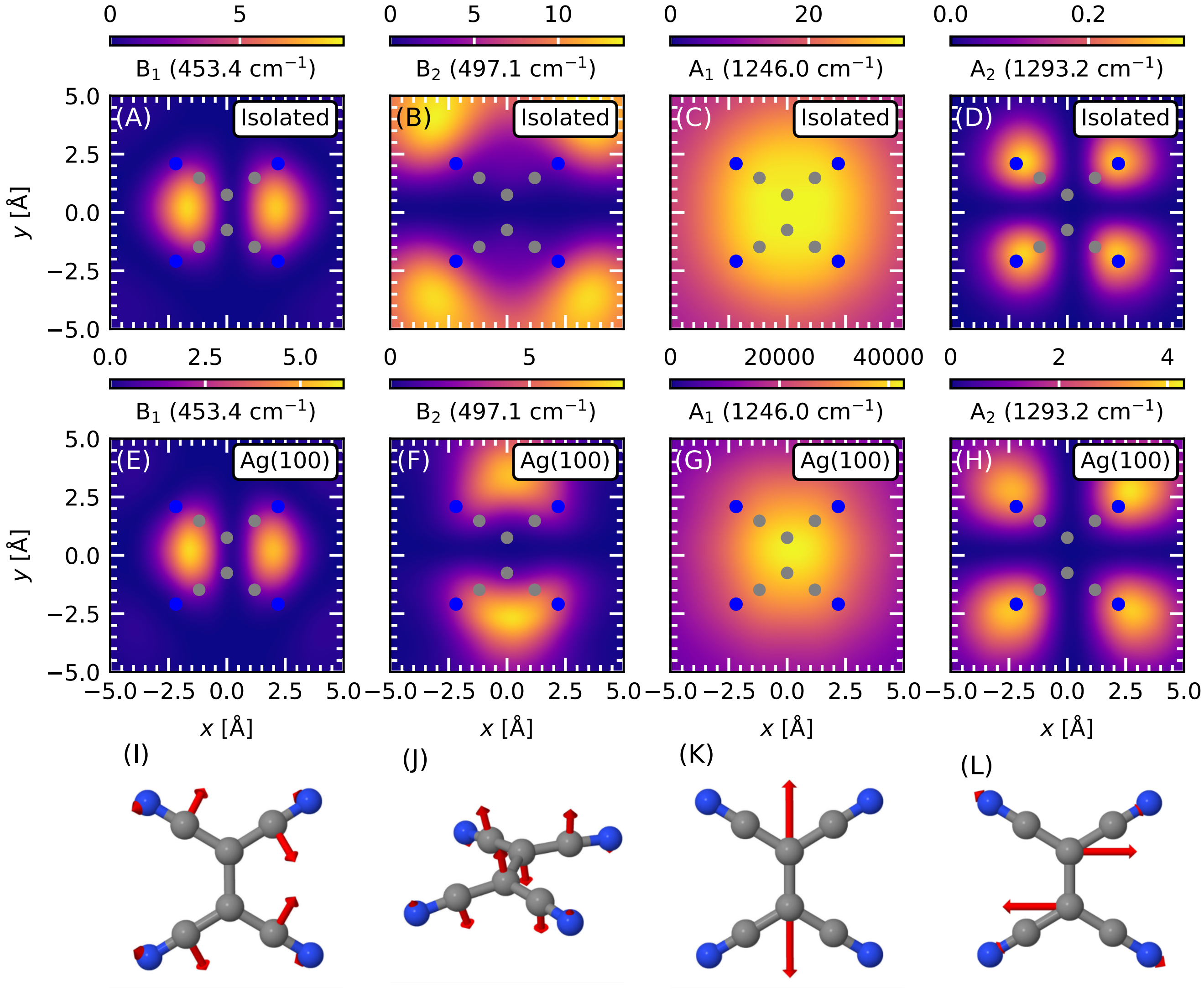

FIG. S5. Simulation of TERS images of selected vibrational modes of TCNE/Ag(100). Panels A–D: Images obtained in the gas phase, however, using displacements calculated on the silver surface. Panels E–H: Images obtained on the explicit silver surface. In all panels A–H the color bars show TERS intensities in the units of 10^4 $e^2 Å^2 V^{-2}$ (see Methods for details). Panels I–L: Snapshots of the corresponding vibrational modes of TCNE/Ag(100). The silver surface was removed for clarity and the red arrows show the atomic components of the Cartesian normal mode vectors. In all panels the following atom color-coding applies: C gray, N blue.

S7. 1D TERS AND FAR-FIELD RAMAN SPECTRA OF THE STUDIED SYSTEMS

In Figure S6, we present the (far-field) Raman and TERS spectra of MoS_2 as a pristine monolayer and containing S vacancies. In Figure S7, we present the Raman and TERS spectra of TCNE/Ag(100) and MgP/Ag(100).

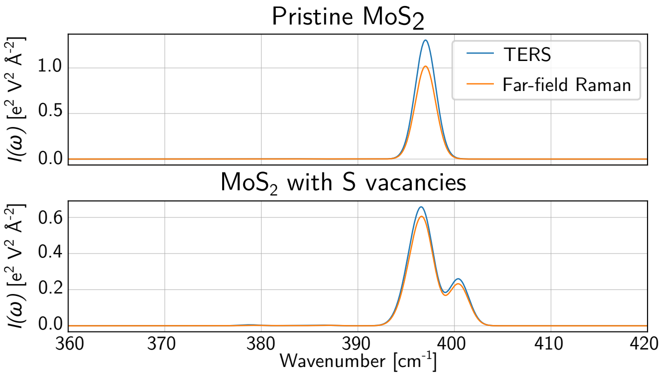

FIG. S6. 1D TERS and far-field Raman spectra of MoS_2. In the case of TERS, the tip was positioned on top of the vacancy defect, or the equivalent position in the pristine system.

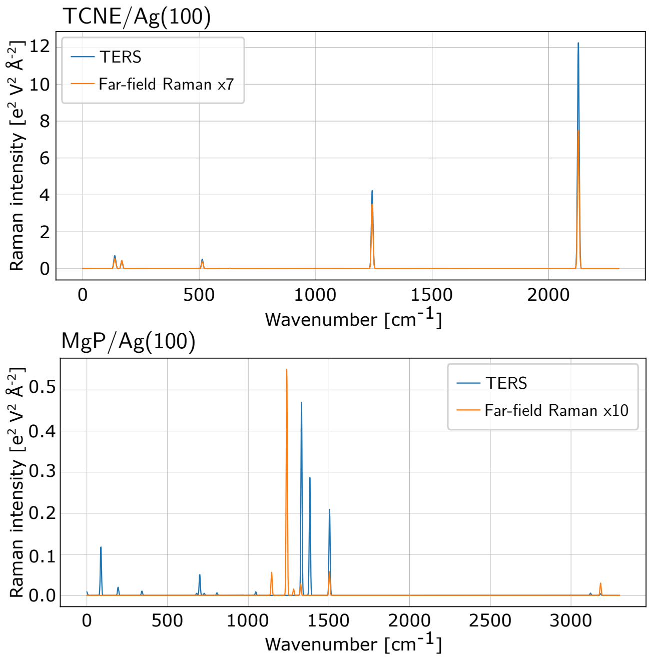

FIG. S7. 1D TERS and far-field Raman spectra of TCNE/Ag(100) and MgP/Ag(100). In the case of TERS, the tip was positioned on top of the molecular center of geometry.

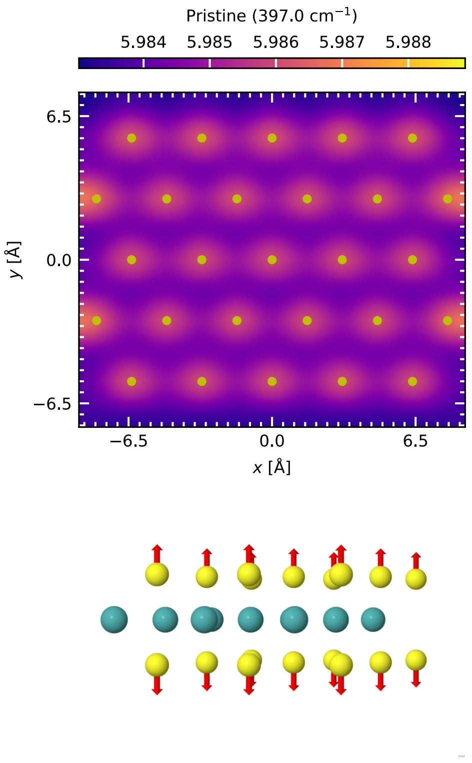

FIG. S8. TERS imaging of the Raman-active A_1' vibration in a pristine MoS_2 monolayer. The top panel shows the calculated TERS image. The positions of the top-layer sulfur atoms are given by the yellow circles. The TERS intensity is shown in the units of 10 $e^2Å^2V^{-2}$. The bottom panel shows a side view the vibration on a 3×3 unit cell. In these snapshots, the sulfur atoms are shown in yellow, the molybdenum atoms in turquoise and the cartesian atomic components of the normal mode vector as red arrows.

S8. PRISTINE MOLYBDENUM DISULFIDE MONOLAYER TERS IMAGING

In Fig. S8, we present the complementary TERS image of the pristine A_1' vibration in MoS_2.

S9. EXPERIMENTAL MG-PORPHINE TERS INTENSITY MAPS

In this Section, we describe our manipulation of the experimental TERS data[S18] of MgP/Ag(100) that led to the plots in Figure 3 of the main text. The starting points were `png` images with a one-to-one correspondence to those shown in Ref. S18, kindly provided by the authors. There was no specification of a color map used to map the experimental intensity to the color shown in those images. Therefore, we identified the best fitting color map to the color spectrum of the images from the `matplotlib` library and then mapped the images to a linear intensity scale normalized to unity. To reach this

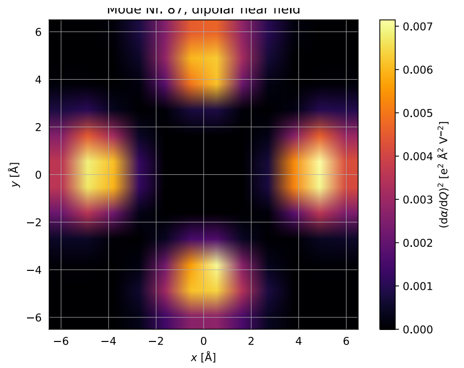

FIG. S9. B_{1g} mode MgP/Ag(100) TERS image calculated with a dipolar field approximating the realistic field used for the production of the results shown in the main text.

goal, we first minimized the norm of the difference between the original RGB value of each pixel of the images and the RGB values of the color map (thus finding the best fitting color) and then relied on the unique and well-defined correspondence between the RGB scale of the chosen color map and the intensity scale. This yielded images that are in excellent agreement with the original images presented in Ref. S18 in terms of their intensity patterns.

We note that original intensity patterns are unit-normalized for each image separately and do not contain absolute TERS intensities. We have further applied a simple rotation-interpolation (using `scipy.ndimage` tools) to the images so that the lobes were aligned with the simulation data. The full spatial extent of the images was accurately determined by the experimentalists; we have merely plotted a sub-region of the images that exactly corresponds to the simulated area of 13×13 Å^2.

S10. IMAGE ASYMMETRY AFTER USING A REALISTIC NEAR FIELD VS. A SYMMETRIC DIPOLAR APPROXIMATION

We have calculated the TERS image of the B_{1g} mode of MgP/Ag(100) calculated with the C_∞-symmetric dipolar-field model of the tip potential that does not break any underlying molecular symmetries. As shown in Fig. S9, using such a near field indeed leads to a symmetric image, within numerical accuracy, in which the four peaks are the same shape and at equivalent positions in comparison to the one obtained with a realistic near field and shown in Fig. 3E of the main text. The remaining (minute) imperfections in the image we understand as a testament to the sensitivity of the finite-field TERS calculations to residual strain from the finite-threshold numerical optimization of the molecular geometry, imprecision in the calculation of normal modes and numerical noise in the integration of the electron density to obtain μ_z.

S11. EFFECT OF TIP SHAPE ON TERS IMAGES

We inspected the influence of the shape and height on the MgP/Ag(100) B_{1g} TERS image. We have compared two tip geometries: "tip A" (shown in Fig. 1 of the main text) and "tip B" and two tip heights of 4 and 5 Å (defined as the apex-to-molecule distance). The geometry of tip B is obtained from tip A by removing the apex atom: as a result, tip B has a flatter, 3-atom apex. These tips have been characterized and discussed previously.[S19] The results of Fig. 1 provide a good gauge of the potential image changes for different tip apexes, which are quantitative but not qualitative.

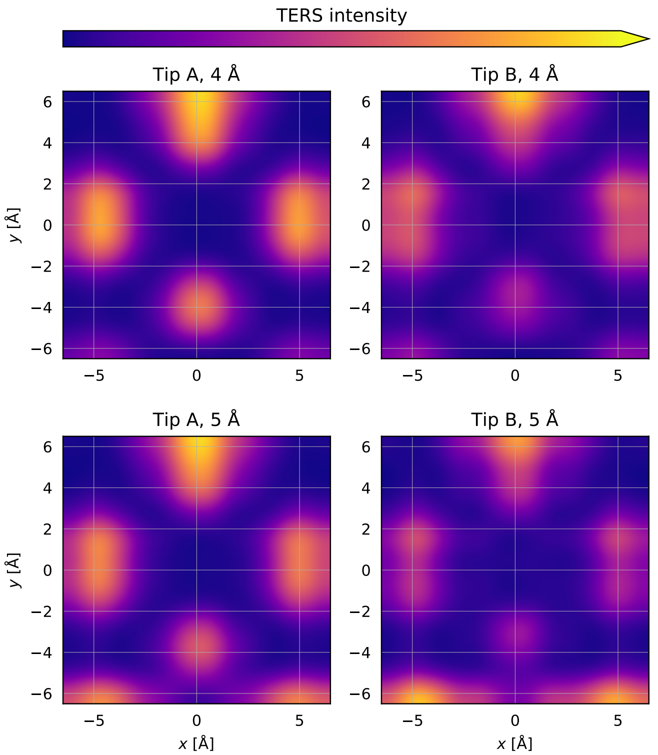

FIG. S10. Comparison of the influence of the shape and height of the employed tip on the TERS image of the B_{1g} mode of MgP/Ag(100). The maximum of the absolute intensity has been unit-normalized in each panel individually.

S12. DEPENDENCE OF THE SHAPE OF TERS IMAGES ON THE EQUILIBRIUM BINDING DISTANCE FROM THE SURFACE

Here, we present a DFT benchmark of the surface–molecule distance and the puckering of the adsorbate for the MgP/Ag(100) system. The puckering is calculated with the so-called total puckering coordinate Q[S21] de-

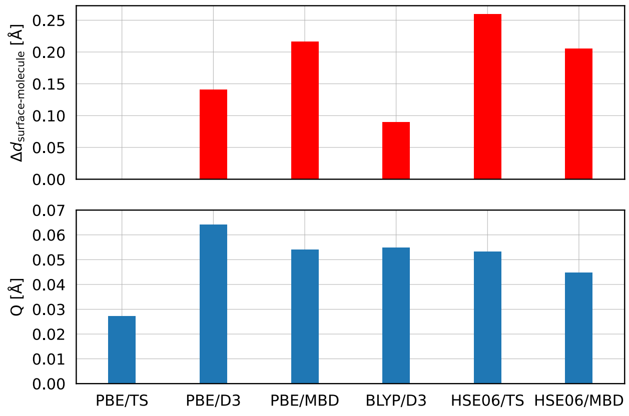

FIG. S11. Description of MgP adsorption geometry on Ag(100) by various DFT functionals and dispersion corrections. Top panel: difference equilibrium binding distance defined as the vertical (z) distance between the average z-position of the first slab layer and the z-coordinate of the MgP center of mass from the PBE/TS reference value. Bottom panel: Projection of the molecular geometry on the total puckering coordinate Q. Note that the MBD-NL method[S20] mentioned in the main text has been shortened to "MBD" in the present plot.

fined as

$$Q = \sqrt{\frac{1}{N_{\text{atoms}}} \sum_{i=1}^{N_{\text{atoms}}} (z_i - z_0)^2}, \tag{S8}$$

where z_i is the vertical components of the positions of the molecule's atoms, z_0 is the z-position of the average plane of the molecule and N_{atoms} is the number of atoms belonging to the molecule.

For all functionals, the puckering remains very small (flat molecule). This, in tandem with the other benchmarks presented in this work, leaves the surface–molecule binding distance one of the very few parameters that can affect the shape of non-resonant TERS images obtained with farther tip-molecule distances, with negligible current. Indeed, as we show in Figure 4 of the main text, changing the dispersion correction from Tkatchenko–Scheffler[S6] to non-local many-body dispersion[S20] can induce a significant change in the TERS image.

S13. CHANGES IN ELECTRON DENSITIES IN MG PORPHINE

In Fig. S12, we demonstrate the origin of the sign change in the quantity A_{zz} discussed in the main text. In the first row, we show the electron density as a function of the z-coordinate

$$\rho(z) \equiv \int_{-\infty}^{\infty} \int_{-\infty}^{\infty} \mathrm{d}x\, \mathrm{d}y\ \rho(x, y, z) \tag{S9}$$

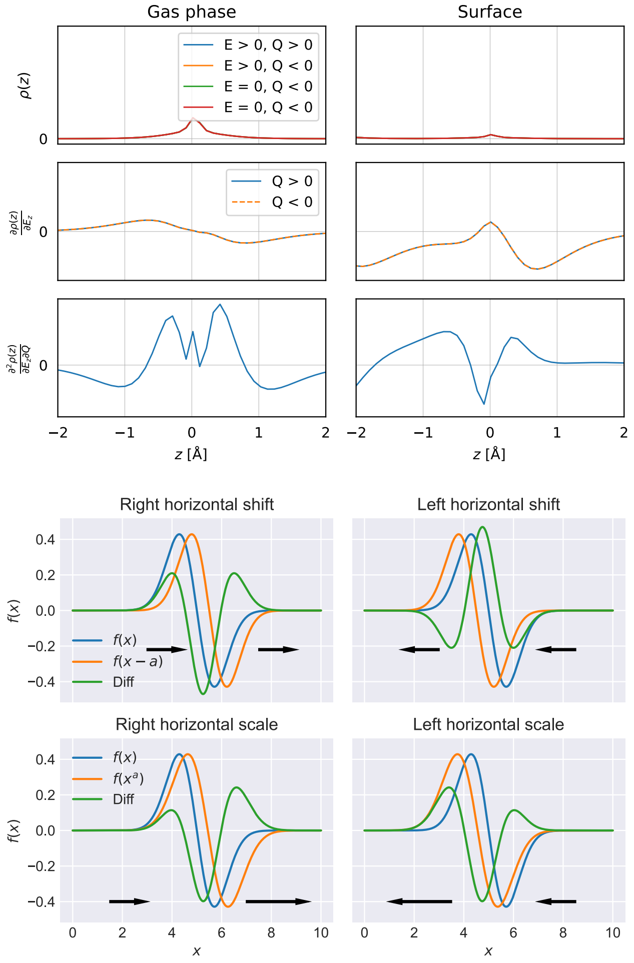

FIG. S12. Top 3×2 grid: the plots of the electron density ρ as a function of the vertical position z, of the density response $\partial\rho/\partial E_z$ and the change of the density response with the molecular vibration $\partial^2\rho/\partial E_z \partial Q_k$. Bottom 2×2 panel: a simple model showing the origin of the $\partial^2\rho/\partial E_z \partial Q_k$ curve shapes. The orange and blue curves represent the function of the general shape $f(x) = -xe^{-x^2}$ with various shifting or scaling transformations; the green curves always shows their difference. The top two panels show that a shift can give rise to the general two-peak pattern, but cannot alone give rise to unevenness in it. The bottom panels show that a horizontal scaling is needed for the unevenness to kick in: this corresponds to a larger electronic polarizability in the realistic case.

with the molecule centered at $z = 0$ for the case with and without the homogeneous far field E_z and at both positive and negative displacements of the normal coordinate Q. In the second row, we show the density response

$$\frac{\partial\rho(z)}{\partial E_z}$$

due to the action of the far field $\mathbf{E} = (0, 0, E_z)$. In the gas phase, this features a clear "beat" pattern corresponding to the polarization of the molecule and the concentration

of negative charge in the $z < 0$ region and of positive charge at the $z > 0$ region. Almost identical pattern is obtained at both displacements $\pm Q$. This observation is qualitatively identical on the surface; however, the negative cloud effectively spills over into the metal, leading to an uneven beat with a more pronounced $z > 0$ side. Finally, in the third row, we plot the second mixed derivative of the density

$$\frac{\partial^2 \rho(z)}{\partial E_z \partial Q_k}.$$

This quantity integrates over z to give A_{zz} and features a two-peak pattern that arises as a horizontal scaling possibly also accompanied by a horizontal shift of the beats at various displacements. In the gas phase, this two-peak pattern is larger at the $z > 0$ side, indicating that the geometry corresponding to $Q > 0$ permits a further displacement of electronic density along the positive direction of z: in other words, the $Q > 0$ is more polarizable in the gas phase. Contrarily, on the surface, the pattern is larger at the $z < 0$ side. This suggests that the $Q > 0$ geometry has its electron density pulled stronger towards the polarized slab that thus screens the change in the density induced by the far field. All in all, this leads to an overall smaller induced dipole in the $Q > 0$ case and, consequently, smaller polarizability and the observed negative A_{zz}.

The bottom panel illustrates the origin of the two-peak patterns observed in the second mixed density derivatives using a simple model for the beat of the form of

$$f(x) = -xe^{-x^2} \tag{S10}$$

centered around $x = 5$. In the first row we show that a simple shift $x \to x - a$ indeed gives rise to the two-peak pattern, but a one that is strictly even, *i.e.*, with both sides of the same height. In order to explain the observed unevenness in the realistic case, one must consider a horizontal scaling $x \to x^{-a}$ that makes the sides uneven. Clearly, this corresponds to the larger or smaller displacements of electron density and the observed differences in polarizabilities. Finally, we note that the region around $z = 0$ does not behave in full accordance with the simple models. This is due to the behavior of the core electrons of the molecule localized in closed proximity of the atoms and is not relevant for the present discussion.

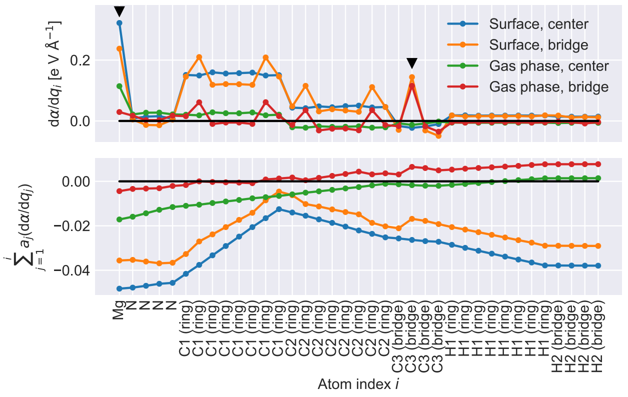

FIG. S13. Atomic decomposition of the TERS amplitude. Top panel: magnitudes of the individual atomic terms. Bottom panel: cumulative sum to show how the overall amplitude for the given tip position is built up from the individual terms. Note that C1 is the pyrrole carbon closer to the center of the molecule, the rest of the labels is self explanatory.

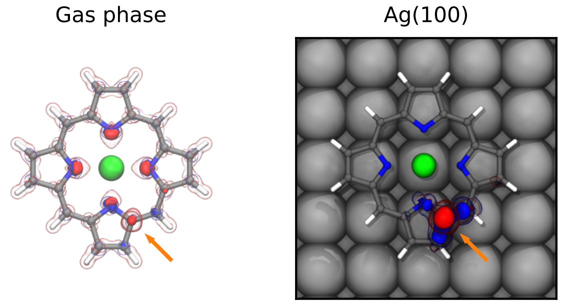

FIG. S14. Spatial dependence of the quantity $\partial^2 \rho(\mathbf{r}; \mathbf{R}_{\rm tip})/\partial E_z \partial z_i$ where the index i corresponds to the pyrrole (C1, see previous Section) carbon atom highlighted by the orange arrow (only the z-displacement is relevant for the A_{2u} mode, *cf.* Fig. 3J). In the gas phase, the shown contours are at ± 1 and $\pm 3 \times 10^{-6}$ e Å^{-3} V^{-1} (red for positive, blue for negative and with the higher isovalue as the solid surface), in the Ag(100)-bound system ± 1 and $\pm 3 \times 10^{-5}$ e Å^{-3} V^{-1}.

S14. ATOMIC DECOMPOSITION OF TERS AMPLITUDE

In the top panel of Fig. S13, we show the individual atomic terms

$$\frac{\partial \alpha_{zz}^{\rm local}(\mathbf{R}_{\rm tip})}{\partial z_i},$$

of the decomposition of the A_{2u} amplitude of MgP in the gas phase and on Ag(100) for two different tip positions. In the above expression, i indexes the atoms in the molecule: note that only the z-direction is relevant for this normal mode. We note that the adsorption makes the terms corresponding to the C2, H1 and H2 atoms change signs and those atoms that are in the closest neighborhood of $\mathbf{R}_{\rm tip}$ give a stronger response. In the bottom panel, we plot the cumulative sum taken over the atomic index i with each term multiplied by the normal mode displacement. As such, at the end of each curve is the full value of A_{zz} in accordance with Equation 2 in the main text.

S15. FURTHER INTERPRETATION OF TERS CROSS TERMS

In this Section, we provide more insight into the balance between self and cross terms of the decomposition of the TERS intensity as discussed in the main text. The question we address here is what exactly the surface does for the cross term to become smaller and less relevant in shaping the surface-bound image? We note that

$$\frac{\partial \alpha_{zz}^{\mathrm{local}}(\mathbf{R}_{\mathrm{tip}})}{\partial q_i} = \int_{\mathrm{unit\ cell}} \mathrm{d}\mathbf{r}\ z \frac{\partial^2 \rho(\mathbf{r}; \mathbf{R}_{\mathrm{tip}})}{\partial E_z \partial q_i} \tag{S11}$$

and inspect the second mixed derivative inside the integrand as a real-space function of $\mathbf{r}$. This quantity (also encountered previously in a slightly different context in Section S13) capture how the electronic density response to a far-field perturbation changes with the molecular vibration. To illustrate its nature, we picked a single atom of the molecule (one of the C1 pyrrole carbons) and moved it from its equilibrium position by $\pm 5 \cdot 10^{-3}$ Å to simulate the variation of q_i and calculate the numeric derivative; the volumetric plot is shown in Fig. S14 for both the gas phase and the surface. While the quantity is fully delocalized over the entire molecule in the gas phase, on the surface we observe a localization to a small surrounding of the plucked atom. This is observed for all atom types in this system. This surface-induced localization could explain the loss of magnitude in the cross term. As shown in Section S14 above, the atomic terms corresponding to atoms located right under the tip apex get enhanced, suggesting that the self terms of atoms in proximity of the tip will be rather strong and the cross terms containing contribution from distant atoms rather weak. We tested this hypothesis for a selected pair of atoms identical to the one shown in Fig S14 (the central Mg and a pyrrole C). The data shown in Figure S15 supports the above claim: the individual atomic terms are maxima localized around the plucked atom, which makes their cross term assume an overall low value as a function of the tip position $\mathbf{R}_{\mathrm{tip}}$ due to their limited overlap. However, there are many more cross terms than self terms, so the outcome will depend on a fine balance of the individual contributions. Testing and analyzing this hypothesis in full would require more extensive calculations of all the individual terms as a function of $\mathbf{R}_{\mathrm{tip}}$.

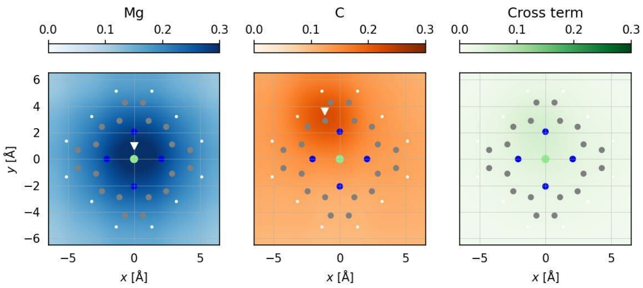

FIG. S15. Left and center: The dependence of the quantity $\partial\alpha_{zz}^{\mathrm{local}}(\mathbf{R}_{\mathrm{tip}})/\partial z_i$ on the tip position $\mathbf{R}_{\mathrm{tip}}$ for the central Mg atom (left, blue) and one of the proximal pyrrole C atoms (center, orange). Both plucked atoms are marked by a white triangle. The unit of the color scale is e V Å^{-1}. Right: the cross product of the two functions in the left and central panel. The unit of the color scale is e^2 V^2 Å^{-2}.

REFERENCES

[S1] Litman, Y.; Bonafé, F. P.; Akkoush, A.; Appel, H.; Rossi, M. First-Principles Simulations of Tip Enhanced Raman Scattering Reveal Active Role of Substrate on High-Resolution Images. *Journal of Physical Chemistry Letters* **2023**, *14*, 6850–6859.

[S2] Abbott, J. W. et al. Roadmap on Advancements of the FHI-Aims Software Package. Roadmap on Advancements of the FHI-aims Software Package. 2025, 2505.00125. arXiv. https://arxiv.org/abs/2505.00125 (accessed Feb 2, 2026).

[S3] Larsen, A. H. et al. The Atomic Simulation Environment—a Python Library for Working with Atoms. *Journal of Physics: Condensed Matter* **2017**, *29*, 273002.

[S4] Blum, V.; Gehrke, R.; Hanke, F.; Havu, P.; Havu, V.; Ren, X.; Reuter, K.; Scheffler, M. Ab Initio Molecular Simulations with Numeric Atom-Centered Orbitals. *Computer Physics Communications* **2009**, *180*, 2175–2196.

[S5] Perdew, J. P.; Burke, K.; Ernzerhof, M. Generalized Gradient Approximation Made Simple. *Physical Review Letters* **1996**, *77*, 3865–3868.

[S6] Tkatchenko, A.; Scheffler, M. Accurate Molecular Van Der Waals Interactions from Ground-State Electron Density and Free-Atom Reference Data. *Physical Review Letters* **2009**, *102*, 073005.

[S7] Litman, Y.; Rossi, M. Multidimensional Hydrogen Tunneling in Supported Molecular Switches: the Role of Surface Interactions. *Physical Review Letters* **2020**, *125*, 216001.

[S8] Hofmann, O. T.; Zojer, E.; Hörmann, L.; Jeindl, A.; Maurer, R. J. First-Principles Calculations of Hybrid Inorganic–organic Interfaces: from State-of-the-Art to Best Practice. *Physical Chemistry Chemical Physics* **2021**, *23*, 8132–8180.

[S9] Neugebauer, J.; Scheffler, M. Adsorbate-Substrate and Adsorbate-Adsorbate Interactions of Na and K Adlayers on Al(111). *Physical Review B* **1992**, *46*, 16067.

[S10] Kohn, W.; Sham, L. J. Self-Consistent Equations Including Exchange and Correlation Effects. *Physical Review* **1965**, *140*, A1133–A1138.

[S11] Broyden, C. G. The Convergence of a Class of Double-Rank Minimization Algorithms 1. General Considerations. *IMA Journal of Applied Mathematics* **1970**, *6*, 76–90.

[S12] Fletcher, R. A New Approach to Variable Metric Algorithms. *The Computer Journal* **1970**, *13*, 317–322.

[S13] Goldfarb, D. A Family of Variable-Metric Methods Derived by Variational Means. *Mathematics of Computation* **1970**, *24*, 23.

[S14] Shanno, D. F. Conditioning of Quasi-Newton Methods for Function Minimization. *Mathematics of Computation* **1970**, *24*, 647.

[S15] Tancogne-Dejean, N. et al. Octopus, a Computational Framework for Exploring Light-Driven Phenomena and Quantum Dynamics in Extended and Finite Systems. *Journal of Chemical Physics* **2020**, *152*, 124119.

[S16] Jensen, L.; Aikens, C. M.; Schatz, G. C. Electronic Structure Methods for Studying Surface-Enhanced Raman Scattering. *Chemical Society Reviews* **2008**, *37*, 1061–1073.

[S17] Miller, J. S. Tetracyanoethylene (Tcne): the Characteristic Geometries and Vibrational Absorptions of Its Numerous Structures. *Angewandte Chemie International Edition* **2006**, *45*, 2508–2525.

[S18] Zhang, Y.; Yang, B.; Ghafoor, A.; Zhang, Y.; Zhang, Y. F.; Wang, R. P.; Yang, J. L.; Luo, Y.; Dong, Z. C.; Hou, J. G. Visually Constructing the Chemical Structure of a Single Molecule by Scanning Raman Picoscopy. *National Science Review* **2019**, *6*, 1169–1175.

[S19] Cirera, B.; Litman, Y.; Lin, C.; Akkoush, A.; Hammud, A.; Wolf, M.; Rossi, M.; Kumagai, T. Charge Transfer-Mediated Dramatic Enhancement of Raman Scattering upon Molecular Point Contact Formation. *Nano Letters* **2022**, *22*, 2170–2176.

[S20] Hermann, J.; Tkatchenko, A. Density Functional Model for Van Der Waals Interactions: Unifying Many-Body Atomic Approaches with Nonlocal Functionals. *Physical Review Letters* **2020**, *124*, 146401.

[S21] Cremer, D.; Pople, J. A. General Definition of Ring Puckering Coordinates. *Journal of the American Chemical Society* **1975**, *97*, 1354–1358.
